# Controlled synthesis of Bi- and tri-nuclear Cu-oxo nanoclusters on metal–organic frameworks and the structure–reactivity correlations†

**DOI:** 10.1039/d1sc05495c

**Published:** 2021-11-29

**Authors:** Qi Xue, Bryan Kit Yue Ng, Ho Wing Man, Tai-Sing Wu, Yun-Liang Soo, Molly Mengjung Li, Shogo Kawaguchi, Kwok Yin Wong, Shik Chi Edman Tsang, Bolong Huang, Tsz Woon Benedict Lo

**Affiliations:** Department of Applied Biology and Chemical Technology, State Key Laboratory of Chemical Biology and Drug Discovery, The Hong Kong Polytechnic University Hong Kong China Twblo@polyu.edu.hk Bolong.huang@polyu.edu.hk; The Hong Kong Polytechnic University Shenzhen Research Institute, The Hong Kong Polytechnic University Shenzhen China; Department of Chemistry, Wolfson Catalysis Centre, University of Oxford Oxford OX1 3QR UK; National Synchrotron Radiation Research Center 101 Hsin-Ann Road Hsinchu 30076 Taiwan; Department of Physics, National Tsing Hua University Hsinchu 30013 Taiwan; Department of Applied Physics, The Hong Kong Polytechnic University Kowloon Hong Kong China; Japan Synchrotron Radiation Research Institute (JASRI), SPring-8 1-1-1 Kouto, Sayo-cho, Sayo-gun Hyogo 679-5198 Japan

## Abstract

Precisely tuning the nuclearity of supported metal nanoclusters is pivotal for designing more superior catalytic systems, but it remains practically challenging. By utilising the chemical and molecular specificity of UiO-66-NH_2_ (a Zr-based metal–organic framework), we report the controlled synthesis of supported bi- and trinuclear Cu-oxo nanoclusters on the Zr_6_O_4_ nodal centres of UiO-66-NH_2_. We revealed the interplay between the surface structures of the active sites, adsorption configurations, catalytic reactivities and associated reaction energetics of structurally related Cu-based ‘single atoms’ and bi- and trinuclear species over our model photocatalytic formic acid reforming reaction. This work will offer practical insight that fills the critical knowledge gap in the design and engineering of new-generation atomic and nanocluster catalysts. The precise control of the structure and surface sensitivities is important as it can effectively lead to more reactive and selective catalytic systems. The supported bi- and trinuclear Cu-oxo nanoclusters exhibit notably different catalytic properties compared with the mononuclear ‘Cu_1_’ analogue, which provides critical insight for the engineering of more superior catalytic systems.

## Abstract 2

The controlled synthesis of novel bi- and trinuclear Cu-oxo nanoclusters supported on UiO-66-NH_2_ that show notably different catalytic properties in the photocatalytic formic acid decomposition reaction is reported.

## Graphical abstract

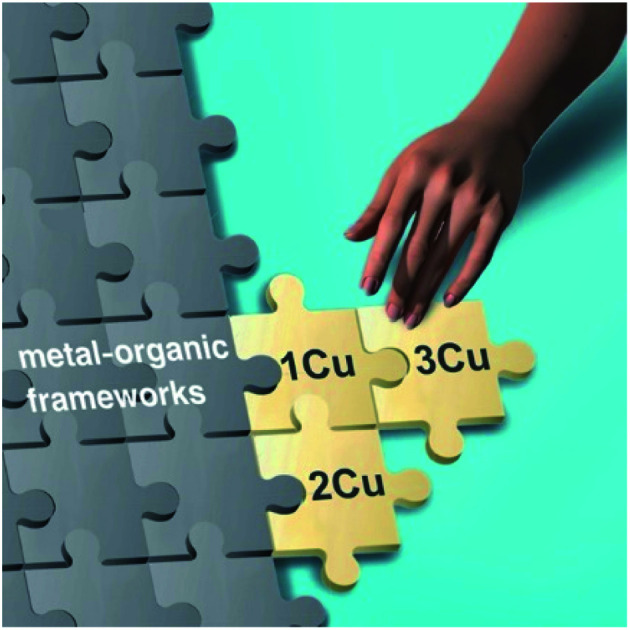

## Introduction

With the global economic boom and population growth, energy conversion has become one of the most important issues in modern society, which often requires the use of catalytic materials. Compared with homogeneous catalysts, heterogeneous catalysts exhibit advantages, such as stability under harsh conditions and facile catalyst-product separation.^1^ Supported single-atoms and nanoclusters (NCs) with low-nuclearity have attracted immense attention in recent years as they effectively bridge between homogeneous and heterogeneous catalytic systems, allowing separation of catalyst from the reaction mixture at the same time as achieving good reactivity.^2,3^ Although single-atom catalysts display attractive characteristics with high atom economy and high surface sensitivity, they are not always the most suitable catalytic active sites.^4^ Meanwhile, NCs often demonstrate remarkably different catalytic properties due to the widely tuneable electronic and geometric characteristics.^5–7^ The chemistry of NCs bridges between bulk (classical chemistry) and atomic/molecular species (quantum chemistry), where the concept of discrete quantum levels in atomic/molecular structures can be fine-tuned.^8^ Understanding the interplay between the structures of active centres, adsorption configuration, catalytic properties, and reaction energetics is pivotal, but addressing this based on experimental evidence remains immensely challenging. The establishment of structure–reactivity correlations between the active centres and catalytic properties is regarded as an important platform for the rational design and engineering of more superior and selective systems. In recent years, various catalytic materials with atomic precision have been fabricated to achieve exciting performance in different applications,^9–11^ such as [Cu_3_(μ-O)_3_]^2+^ supported on MOR zeolite for the selective oxidation of methane to methanol by Lercher *et al.*,^12^ and the engineering of MIL-101(Ti)-based Cu_2_ species as artificial monooxygenase by Lin *et al.*^13^

The catalytic characteristics of NCs can generally be correlated to nuclearity (number of atoms),^14,15^ electronic (HOMO–LUMO work functions and binding affinities)^16^ and geometric properties (morphology and coordination environments)^17^ of the active sites. Some reaction mechanisms are governed by the adsorption configurations of the substrates.^18,19^ We, however, still cannot accurately predict the effect of these parameters on catalysis, which greatly hinders the precise engineering of this class of highly potent and atom-economical catalysts.

A notable example is the catalytic reforming of formic acid (HCOOH, ‘FA’); note that FA is regarded as a ‘liquid’ hydrogen carrier, alongside other small molecules such as methanol and ammonia. Conventionally, FA reforming is realised through an acidic condition that requires the use of concentrated sulphuric acid. Corrosion and reusability problems prevent it from the real application.^20^ The catalytic FA reforming, over solid-state materials by photo-, electro-, and thermal approaches, has hence attracted recent research attention. It is reported that the product distribution of FA reforming is governed by the surface structures of the active sites.^21–23^ Briefly, there are two main proposed pathways for FA reforming, namely, dehydration and dehydrogenation pathways, which have been found dependent on the above-discussed characteristics. Based on various theoretical findings, the dehydrogenation pathway (HCOOH → CO_2_ + H_2_) is favoured when FA is bridged on two adjacent metal sites on a flat terrace surface, whereas both dehydration (HCOOH → CO + H_2_O) and dehydrogenation pathways are competing mechanisms when FA is adsorbed on an isolated metal site. Depending on the surface structures, FA molecules can either be adsorbed *via* monodentate (activated formate *COOH) or bidentate (activated carboxyl *HCOO) mode. Following monodentate adsorption, the activated *COOH species can be reformed *via* competing C–O cleavage to give CO or *via* O–H activation to give CO_2_. In contrast, following bidentate adsorption, the activated *HCOO species preferentially gives CO_2_ from C–H activation. The product selectivity is governed by the energetics of specific reactive surfaces.^24,25^ This reaction has been widely used to investigate the interplay between surface structures and catalytic reactivities.^26^

Herein we report the controlled synthesis of precise bi- and trinuclear Cu-oxo (Cu_2_- and Cu_3_-) active centres stabilised by the Zr_6_O_4_ nodes of UiO-66-NH_2_ (a Zr-based metal–organic framework (MOF)) by exploiting the underlying principles of coordination chemistry and solid-state chemistry. The detailed structural parameters and crystal structures are elucidated by combined X-ray absorption spectroscopy (XAS) and Rietveld refinement of high-resolution synchrotron X-ray powder diffraction (SXRD). By further studying their corresponding catalytic performance on the model photocatalytic FA reforming reaction with respect to the mononuclear (Cu_1_) analogue, the structure–reactivity correlations are revealed at atomic resolution.

## Results and discussion

**Fig. 1:**
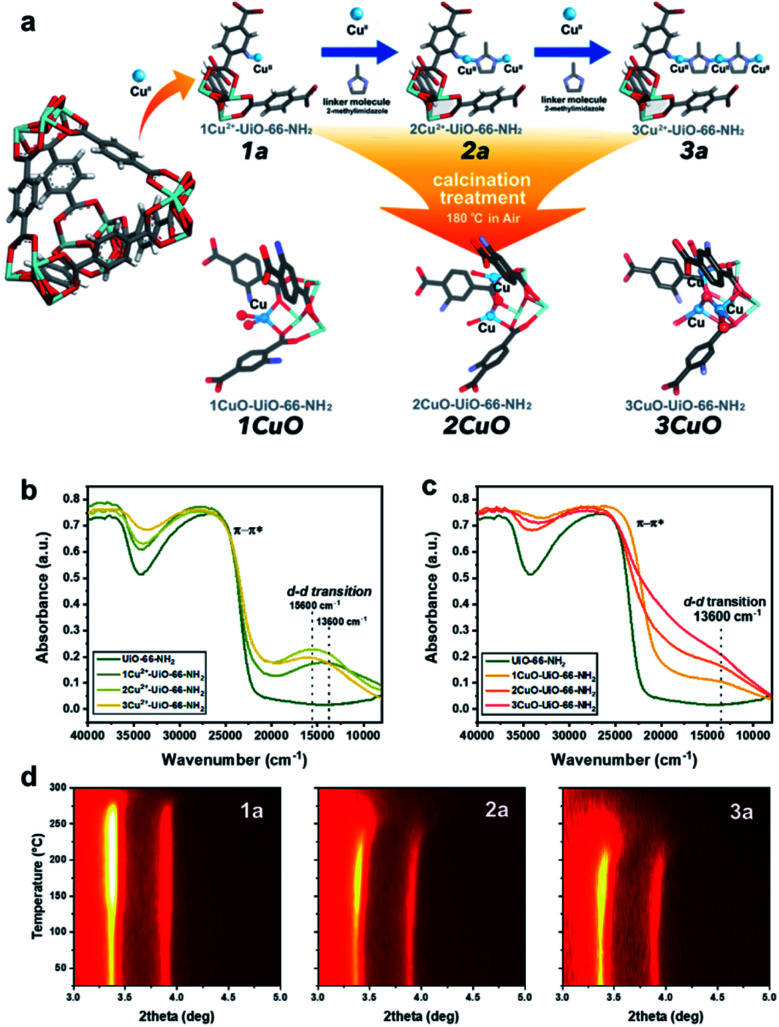
(a) The proposed model of the controlled synthesis procedure of 1a, 2a, 3a, 1CuO, 2CuO, and 3CuO. Coordinated waters and ligands are omitted for clarity. Atoms are presented in balls-and-sticks model. Colour scheme: Zr = light blue, O = red, C = grey, N = purple, H = white, and Cu = blue. UV-vis-NIR diffuse reflectance spectra of (b) UiO-66-NH_2_, 1a, 2a, and 3a; (c) 1CuO, 2CuO, and 3CuO. Temperature-resolved PXRD 2-D contour plot of the calcination treatment *in situ* of (d) 1a, 2a, and 3a.

A crystalline UiO-66-NH_2_ powder sample as solid-state support has been prepared (see Fig. S1 and S2 in ESI†) with a chemical formula of Zr_6_O_4_(OH)_4_ (CO_2_C_6_H_3_NH_2_CO_2_)_5.7_, with a Zr : linker ratio of 1 : 0.96. We first employed bis(ethylenediamine)copper(ii) hydroxide as the Cu^II^ precursor for metalation to form 1Cu^2+^-UiO-66-NH_2_ (denoted as ‘1a’ for simplicity’). As shown in our previous work, the Cu^II^ site is anchored on the framework –NH_2_ site *via* Lewis acid–base interaction with a vacant water site.^27^ Excess Cu^II^-metalation steps have been taken to ensure the maximum capability of UiO-66-NH_2_ has been reached. As shown in Fig. 1a, we further utilise the di-basic chemical and geometric specificity of 2-methylimidazole (meIm) to form a Cu^II^-imidazole complex. This results in an open basic N-site on the imidazole, which can further deprotonate and ‘link’ another Lewis acidic Cu^II^ reagent to form a bi-copper complex structure, 2Cu^2+^-UiO-66-NH_2_ (‘2a’). Similarly, we can synthesise a tri-copper complex structure, 3Cu^2+^-UiO-66-NH_2_ (‘3a’), in a modular manner. The samples were then calcined at 180 °C to yield the final ‘Cu-oxo’ catalysts, namely, 1CuO- (‘1CuO’), 2CuO- (‘2CuO’), and 3CuO-UiO-66-NH_2_ (‘3CuO’).

As shown in the elemental analysis in Table S1,† the atomic ratio of Cu : Zr increases linearly from 0.09 to 0.21 to 0.30 for 1a, 2a, and 3a, respectively, rendering a ratio of nearly 1 : 2 : 3. Non-100% uptake of metal species by the linker functionality is common due to charge balance limitation. In our control experiment, we do not observe any increase in the Cu content without the application of the meIm linker, as the initial metalation capacity has already been saturated at the given condition. The elemental analysis suggests that the application of meIm evidently leads to an increase in the Cu content.

It is critical to analyse whether our assembly approach has led to the formation of the bi- and trinuclear complex structures, instead of forming additional ‘isolated’ Cu^II^ sites on the framework –NH_2_. We accordingly employed UV-vis-NIR diffuse reflectance spectroscopy to analyse the Cu^II^–meIm interaction (see Fig. 1b). Upon the addition of meIm, it shows a hypsochromic shift in the d–d transition, from *ca.* 13 600 cm^−1^ in 1a to *ca.* 15 600 cm^−1^ in 2a and 3a. This can be attributed to the stronger Cu–N_meIm_ interaction than the original Cu–O_water_ interaction as meIm is a strong σ donor and a weak π acceptor ligand. Clearly, the hypsochromic shift indicates Cu–N_meIm_ interaction. On the other hand, the d–d transitions of 2a and 3a are comparable, which can reflect that the Cu^II^ species are in similar chemical environments. Another point to note is the increase in the intensity of the peak reflects that the Cu^II^ concentration in 3a is higher than those in 1a and 2a, which agrees with our elemental analysis.

We further examined the structural properties of 1a, 2a, and 3a by studying their coordination environments using extended X-ray absorption fine structure (EXAFS) spectroscopy. As seen in the Fourier transforms of the EXAFS *k*-space data (Cu K-edge) in Fig. S3,† there is only one prominent peak in the 1 Å < *r* < 2 Å region that matches with the Cu–N/O scattering path at *ca.* 1.5 Å. It should be noted that Cu–O and Cu–N are barely distinguishable by typical X-ray techniques because of the proximity in their scattering factors. From our fitting results (Table S2†), the Cu–N/O bond lengths are calculated as *ca.* 1.95 Å with a Cu–N/O coordination number of *ca.* 4. Very weak backscattering and lack of long-range Cu–Cu scattering paths are noted beyond 2 Å, suggesting the absence of metal sintering. Rietveld refinement of high-resolution SXRD and density functional theory (DFT) calculations have been combined to study the crystal structures (see experimental and computational details in methods/ESI†). The crystallographic parameters and refinement profiles are presented in Fig. S4–S7 and Table S3.† It is apparent that the void space within UiO-66-NH_2_ (void aperture *ϕ* of *ca.* 11 Å) can host the relatively bulky bi- and trinuclear complex structures. The corresponding formation energy has also been calculated, which decreases rapidly from 3.65 eV to 0.25 eV for 1a to 3a (Fig. S8†). The formation energy of a hypothetical ‘4Cu^2+^-UiO-66-NH_2_’ is nearly 0 eV, which agrees with our experimental attempt that it cannot be synthesised based on elemental analysis. The contrasting influence between enthalpy and entropy can be clearly seen, in which the gain in enthalpy in the fourth Cu-metalation step is unable to compensate for the entropy loss.

The calcination condition at 180 °C in the air was chosen to prepare the corresponding MOF-based Cu-oxo samples, as some N-containing ligands (such as ethylenediamine) on Cu(ii)-based complexes were shown removed at this condition.^28^ We have accordingly evaluated this condition by TGA (Fig. S9†) and *in situ* powder X-ray diffraction (PXRD, see Fig. 1d, S10 and S11†), which shows that there is a significant structural change at around 150 °C. From the shape of the *in situ* PXRD profiles, a surge in (111) and (200) is observed at around 150 °C in all samples, which can be attributed to the removal of guest species from the macropore.^29^ Another apparent feature from the *in situ* PXRD is the change in the thermal stability, in which the decomposition temperature decreases consistently from about 275 °C (1a) to 250 °C (2a) to 225 °C (3a). This reveals a substantial difference in the stability of the crystal structures where the incorporation of Cu moieties in 3a could influence the host UiO-66-NH_2_ the most. In addition, we have evaluated the TGA results to extrapolate the quantity of meIm per Cu site in our samples. As shown in the detailed calculation in the ESI,† the Cu : meIm ratios for 2a and 3a are calculated to be 2.18 : 1 and 1.44 : 1, respectively, which tends to the atomic ratios of 2 : 1 and 3 : 2. This renders solid evidence about the bi- and trinuclear complex structures, as illustrated in Fig. 1: in 2a, 2 Cu^II^ nuclei are held by 1 meIm; in 3a, 3 Cu^II^ nuclei are held together by 2 meIm.

From the above characterisation, it is evident that these single atoms, bi- and trinuclear complexes are sparsely (*ca.* 0.1 Cu-based entity per framework –NH_2_ group) but evenly distributed (thermodynamic distribution, about 10.3 Å apart from the crystallographic perspective) within the UiO-66-NH_2_ macropore. Upon calcination at 180 °C, these Cu-based entities should not aggregate as no peak broadening or any formation of new Cu or CuO crystalline phases are observed from the *in situ* PXRD results. Similarly, the electronic properties can be probed by UV-vis-NIR diffuse reflectance spectroscopy. As presented in Fig. 1c, the spectroscopic features are changed substantially upon calcination. Notably, a substantial peak shouldering from the peak at *ca.* 26 500 cm^−1^ (assigned to π–π* of the MOF linker) is noted. This can be attributed to the alternation of the electronic structure through ligand-to-metal charge transfer of the linker by direct interactions with extra-framework Cu-based species, such as Cu anchoring on the Zr_6_O_4_ nodal centre.^30^ Meanwhile, a d–d transition of Cu^II^ at 13 600 cm^−1^ can be observed from the calcined samples (1CuO, 2CuO, and 3CuO). The d–d transition of Cu^II^ is returned from 15 600 cm^−1^ to the initial value at 13 600 cm^−1^ as observed in 1a, which suggests the removal of the meIm linker. The high-resolution scanning transmission electron micrographs are presented in Fig. S12–S14.† We note no observable formation of surface nanoparticles. The UiO-66-NH_2_ framework is retained upon Cu^II^-metalation as shown by Raman spectroscopy (Fig. S15†). The apparent optical band gaps of the samples have been extrapolated using UV-vis spectroscopy (Tauc plot), where it decreases from 2.71 eV (for UiO-66-NH_2_) to 2.66 ± 0.01 eV (for 1CuO, 2CuO, and 3CuO) (Fig. S16†).

The bonding interactions of the three Cu-oxo samples have been characterised by Fourier transform infrared spectroscopy (FTIR) (Fig. S17†). The peaks at around 560 cm^−1^ and 650 cm^−1^ can be ascribed to Zr–(O–C) asymmetric and symmetric stretching modes of the MOF framework.^31^ Noticeable changes in peak positions and the peak intensities can be observed upon calcination, suggesting a change in Zr–O bonding from the direct interaction of the Zr_6_O_4_ nodal centre with the Cu moieties.^31,32^ A broad peak at around 3670 cm^−1^, which is assigned to the O–H stretching of Cu(μ_2_-OH)Cu species, is also observed after calcination, which indicates the formation of Cu(oxo) cluster.^11,33^ From X-ray photoelectron spectroscopy (Fig. S18†), we observed a bathochromic shift of Zr-3d from the addition of Cu, reflecting an increase in the electron density. Meanwhile, the Zr-3d energy in 2CuO and 3CuO is slightly lower than that in 1CuO, which suggests the promoted electronic interaction between Cu and Zr in proximity.^34^

**Fig. 2:**
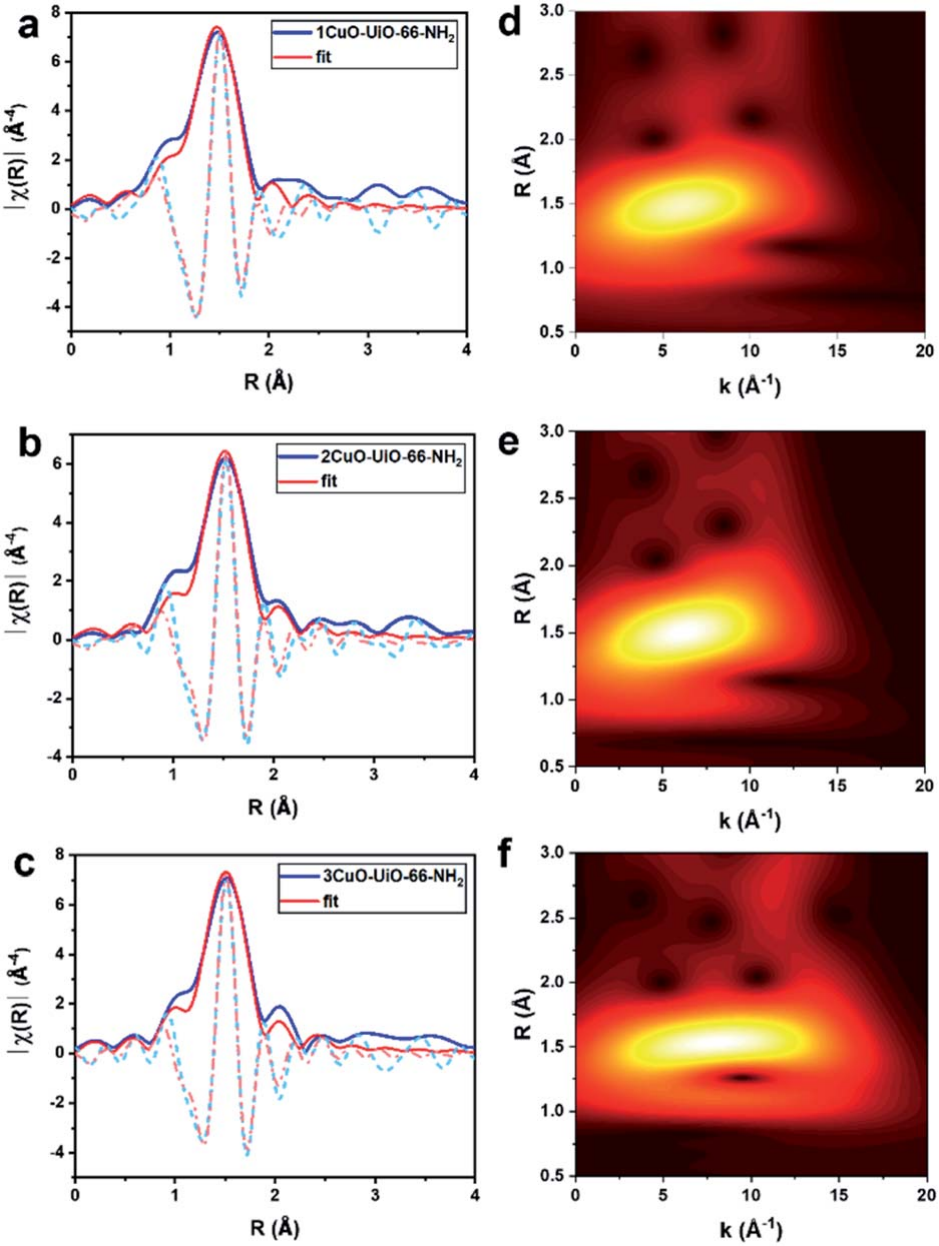
Fourier-transformed magnitude of the experimental Cu K-edge *k*^3^-weighted EXAFS data and fitting profiles of (a) 1CuO, (b) 2CuO, and (c) 3CuO. Wavelet transforms for the EXAFS signals of (d) 1CuO, (e) 2CuO, and (f) 3CuO.

X-ray absorption spectroscopy (XAS) has been accordingly employed to discern the local electronic and geometric structures of the Cu-oxo samples. The Cu K-edge X-ray absorption near-edge structures of 1CuO, 2CuO and 3CuO are shown in Fig. S19.† The pre-edge feature at 8978 eV is assigned to the 1s → 3d transition of typical Cu^II^ species. The oxidation state of 2+ can be confirmed in the Cu-oxo samples by comparison with the reference spectra of CuO and Cu_2_O. The coordination environments of the Cu^II^ species in 1CuO, 2CuO and 3CuO are different from that of CuO, as shown in the difference between the X-ray near edge spectroscopy (XANES) results. This suggests that the Cu^II^ species do not adopt a square planar symmetry as observed in typical Cu^II^ compounds like CuO, but they feature pseudo tetrahedral geometry.^35^ The change in symmetry is often driven by the steric effect; [Cu(H_2_O)_6_]^2+^ (*D*_4h_) becomes [CuCl_4_]^2−^ (nearly tetrahedral) upon Cl^−^ substitution.^36–38^ The coordination information of the Cu centres can be obtained by the quantitative fitting of the EXAFS data (*R*-space in Fig. 2a–c, and *k*-space in Fig. S20†). An obvious peak at *ca.* 1.47 Å can be assigned to the Cu–O path. As summarised in Table S4,† for all three samples, the quantitative fitting results show a Cu–O bond distance of *ca.* 1.95 Å with a coordination number between 3–4.

To better correlate the EXAFS peaks with *k*-space, wavelet transformation (WT) was employed (Fig. 2d–f, the zoom-in of the *R*-space between 2–3 Å are shown in Fig. S21†) using the software package developed by Funke and Chukalina using Morlet wavelet with *κ* = 10, *σ* = 1.^39^ In 1CuO, the highest intensity belongs to the lobe centred at *k* = 6.5 Å^−1^, *R* = 1.5 Å, which corresponds to the O atoms around the Cu centre. Some lobes with weak intensity can also be observed at *R* > 2.0 Å, which can be attributed to the backscattering of Cu⋯Zr_framework_ interaction. We can see that the WT-EXAFS of 1CuO has a notably different topology compared with those of 2CuO and 3CuO. In particular, the lobe at around *k* = 10.0 Å^−1^, *R* = 2.0 Å is further extended in 2CuO and 3CuO, which suggests the existence of second-shell Cu⋯Cu interactions. In general, the contribution of heavier atoms, such as Cu in our system, tends to stretch the lobes towards higher *k*-space.^40^

**Fig. 3:**
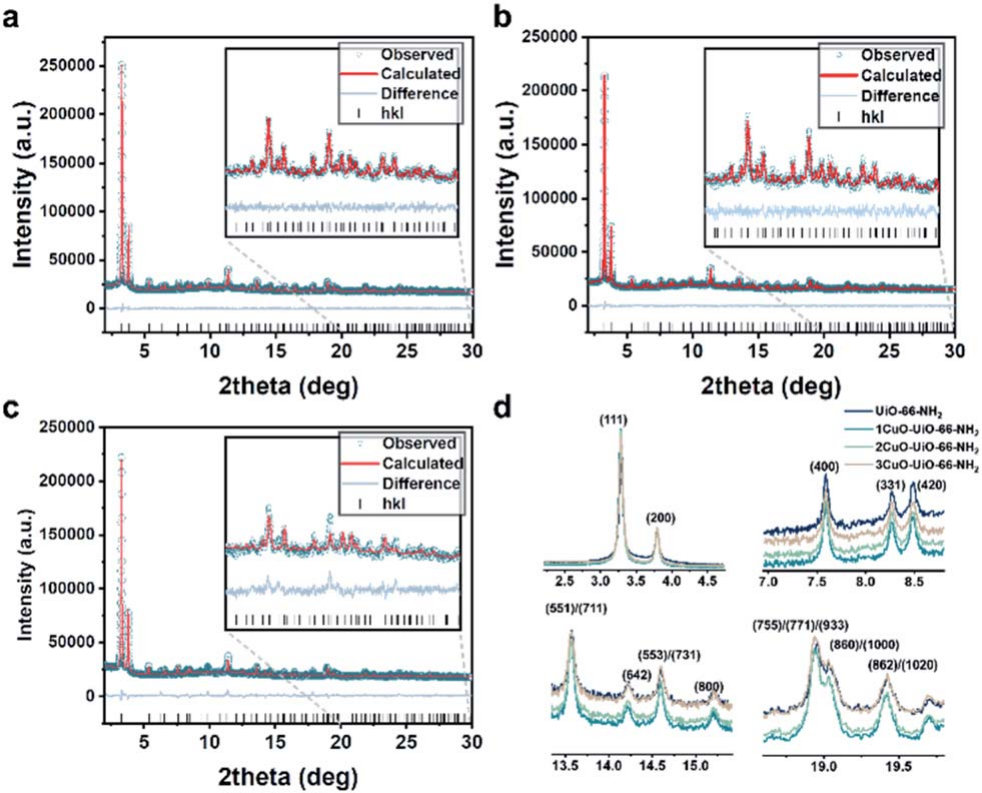
High-resolution SXRD patterns collected on beamline BL02B2 at SPring-8 (*λ* = 0.688213(10) Å; *E* = 18 keV) and the Rietveld refinement profiles using TOPAS v6.0 of (a) 1CuO, (b) 2CuO, and (c) 3CuO. The crystallographic parameters are summarised in Table S6.† (d) Normalised data (using *hkl* = 200) showing significant variation between the diffraction patterns. A quantitative comparative analysis of the peak intensity is shown in Table S7.†

**Fig. 4:**
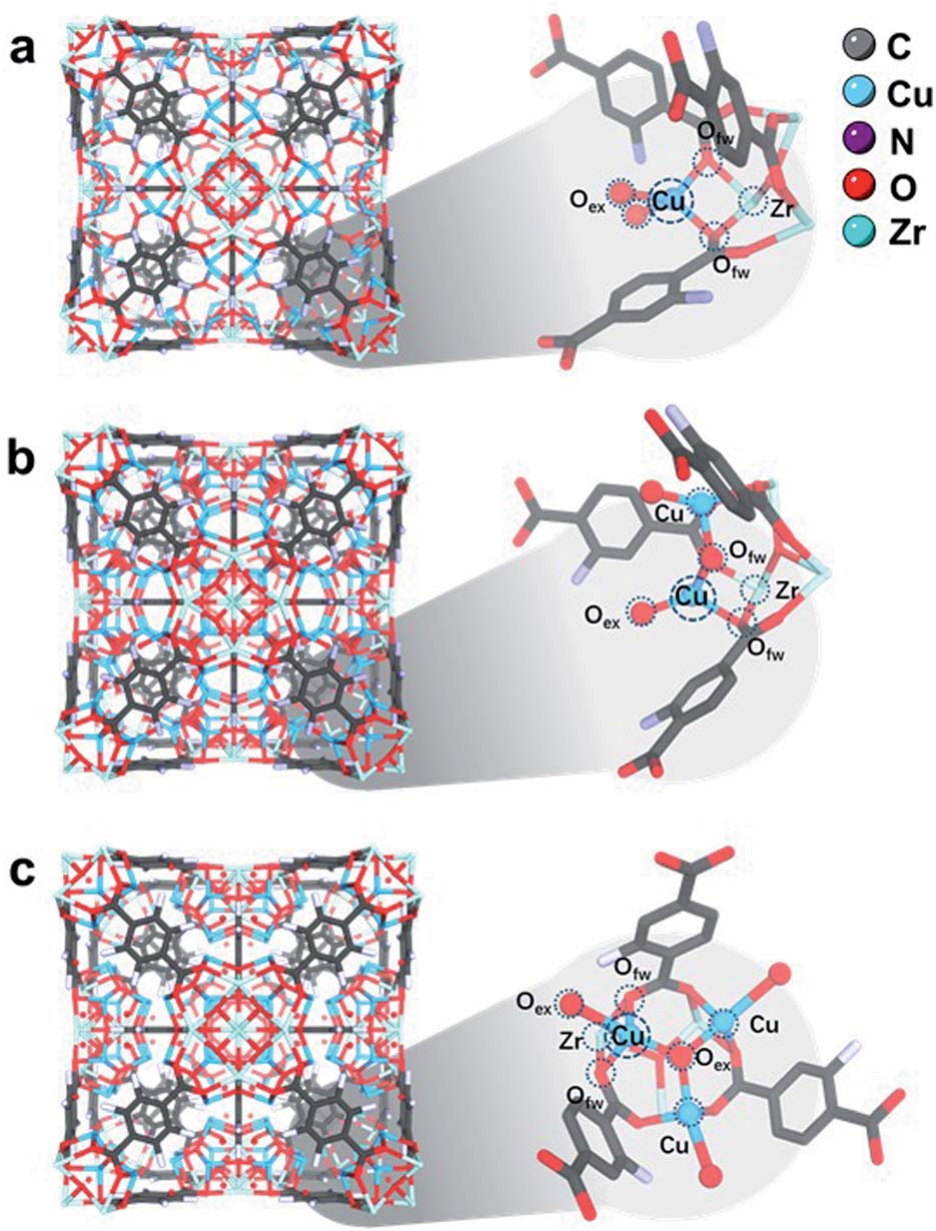
Rietveld refined crystal structures of (a) 1CuO, (b) 2CuO, and (c) 3CuO. Zoom-in images of the asymmetric unit are shown, showing the interaction between Zr–O metal nodes and Cu atoms. The Fourier electron maps are shown in Fig. S26.† The detailed atomic parameters are summarised in Table S9.† For better visualisation, a clearer illustration of the crystal structures is shown in Fig. S27.†

The high-resolution SXRD patterns of 1CuO, 2CuO, and 3CuO are presented in Fig. 3a–c. A variation in the Bragg peaks' intensities can be seen in Fig. 3d. The positions of the Bragg peaks only changed marginally (space group: *Fm*3̄*m*), suggesting that the extra-framework Cu species did not alter the crystalline framework of the host UiO-66-NH_2_. The crystallographic parameters of the samples are summarised in Table S5,† in which a slight expansion of the lattice is noticed upon the incorporation of Cu species. The unit cell parameters increase to a different extent, where 1CuO shows an increase of 0.21% compared with a smaller increase of 0.18% for 2CuO and 0.06% for 3CuO. Apparent changes in Bragg peak intensities in the higher 2*θ* regime upon Cu metalation can be attributed to the significant changes in the scattering factors which arose from heavier atoms. Through the quantitative analysis of the SXRD patterns, the distribution of Cu species can be probed. As presented in Fig. S22 and Table S5,† the Bragg peaks remain highly symmetrical in all the samples, suggesting that the metalation process is highly homogeneous. The Rietveld refined crystal structures of the Cu-oxo samples are presented in Fig. 4. High-quality of the Rietveld refinement can be confirmed by a low *R*-weighted pattern (*R*_wp_) value and a small difference between the data and the fitting profiles. The site occupancy factors of the Cu sites have been fixed on the basis of the elemental analysis results to ensure the reliability of the Rietveld refinements.

In the refined structure of 1CuO, Cu1 is directly immobilised on the Zr_6_O_4_ nodal centre, with derived bond lengths of 1.94(1) Å for both Cu1–O1_f_ and Cu1–O1_f′_. Cu1 is also coordinated with two non-framework oxygen atoms, with Cu1–O1_w_ and Cu1–O2_w_ = 1.97(2) Å. The Cu1 site adopts a pseudo-tetrahedral geometry presumably due to framework rigidity and steric hindrance. In that of 2CuO, Cu1 and Cu2 are immobilised on Zr_6_O_4_ with the derived bond lengths of 2.01(1) Å for Cu1–O1_f_, and Cu2–O2_f_. The two Cu sites are bridged together by μ_2_-O_μ_ with Cu–O bond lengths of 1.86(1) Å. Similarly, the Cu sites adopt pseudo-tetrahedral geometry and are also coordinated with extra-framework O_w_ atoms with bond distances of 1.95(2) Å. In that of 3CuO, Cu1, Cu2 and Cu3 are immobilised on Zr_6_O_4_ with derived bond lengths of 2.01(1) Å for Cu1–O1_w_, Cu2–O2_w_, and Cu3–O3_w_. The Cu sites are bridged together by μ_3_-O_μ_ with bond lengths of 1.95(1) Å. It is noted that the non-framework oxygen atoms are often present in the form of a terminal –OH instead of –OH_2_ to maintain charge neutrality.^41,42^ The refinement profiles without the inclusion of Cu-oxo species are compared in Fig. S23–S25 and Table S8.† Clear differences can be seen which show the notable contribution of the Cu-oxo species in the Rietveld refined crystal structures.

We found that these Cu sites in the refined crystal structures all adopt pseudo tetrahedral geometries, based on the derived bond angles and distances. This is in great agreement with our XANES results and previous Cu^II^-imidazolate complex study.^38^ To better verify the accuracy of the refined crystal structures, we have analysed the simulated EXAFS and WT-EXAFS profiles (Fig. S28–S30†).

**Fig. 5:**
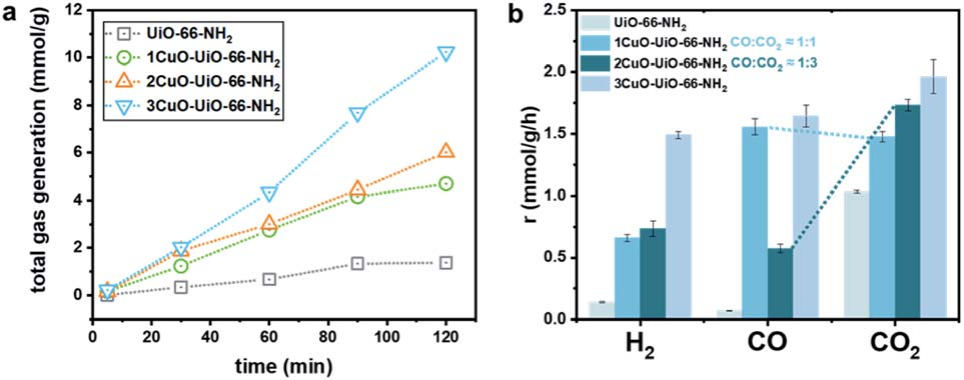
(a) Total gas generated from photocatalytic reforming of FA over the UiO-66-NH_2_ and Cu-oxo catalysts, and (b) the corresponding product distribution of H_2_, CO, and CO_2_.

Apparently, we have synthesised novel bi- and trinuclear NCs (Cu_2_-oxo and Cu_3_-oxo) that are stabilised by the Zr_6_O_4_ node of UiO-66-NH_2_. The atomic arrangements of the active sites are notably different compared with the single-atom counterpart. A major disparity is the nuclearity of Cu atoms at the active sites. As mentioned earlier, the catalytic reforming of FA is partly governed by the adsorption geometry. We thereby investigate how the nuclearity of the Cu-oxo active sites affects the adsorption configuration and its reactivity *via* the model photocatalytic reforming of FA. The photocatalytic reforming of FA was performed using a 500 W Xe lamp with an approximate luminance of 400 cd mm^−1^ (see experimental details in ESI†/methods). The main products in the gaseous phase are H_2_, CO, and CO_2_. No product in the liquid phase has been noted (Fig. S31†). The catalytic product distribution is summarised in Fig. 5. Some by-products are also detected, such as from the side reaction of the hydrogenation of CO to methane (<1% detected).^43,44^ From our time-resolved (Fig. 5a) and recycling (Fig. S32†) experiments, only a slight fluctuation in the catalytic properties is observed. The crystal structure remains highly crystalline and does not show other crystalline phases post reactions (see Fig. S33†).

As seen in Fig. 5a, the total gas evolution over the Cu-oxo catalysts is much higher than that over pristine UiO-66-NH_2_, suggesting that the Cu-oxo active sites play pivotal roles in the reaction. The total gas evolution over 1CuO is comparable to that over 2CuO, as shown in Fig. 5b. For 1CuO and 2CuO, their CO and CO_2_ productions are noticeably different, where 1CuO produces a higher concentration of CO compared to CO_2_ (*cf.* 1.56 and 1.48 mmol g^−1^ h^−1^), whereas 2CuO produces a lower concentration of CO compared to CO_2_ (*cf.* 0.58 and 1.73 mmol g^−1^ h^−1^). This gives contrasting CO : CO_2_ ratios of 1.05 : 1 *versus* 0.33 : 1. Alternatively, the production of both CO and CO_2_ increases for 3CuO with respect to 2CuO, where the CO : CO_2_ ratio was determined as 0.84 : 1. Such distinct product distribution implies that different reaction pathways are involved.

As seen in the UV-vis results (Fig. S16†), the apparent optical band gaps of 1CuO, 2CuO, and 3CuO were determined as 2.66 ± 0.01 eV. The difference is insignificant, suggesting that the variation in the product distribution of FA reforming should predominantly be caused by two key parameters, namely, the structures of the active sites and the energetics of the reaction. As mentioned, there are two major pathways for FA reforming reaction, dehydration (HCOOH → CO + H_2_O) and dehydrogenation (HCOOH → CO_2_ + H_2_), that are highly related to the adsorption modes (monodentate formate or bidentate carboxyl) between the FA molecule and the surface structures of the active sites.

**Fig. 6:**
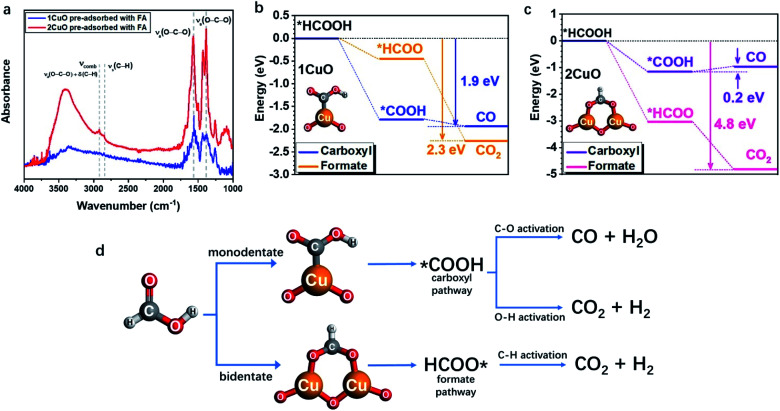
(a) FTIR spectra of 1CuO and 2CuO with pre-adsorbed FA. The overall reaction energy trends of (b) 1CuO and (c) 2CuO. (d) The proposed reaction pathways of FA reforming, showing the interplay between adsorption configuration and pathway selection.^24,25,51^

As the structural disparity between 1CuO and 2CuO is the most profound, we employed FTIR to investigate the difference in their adsorption configurations of FA (Fig. 6a). Major features have been observed at around 1380 cm^−1^ and 1570 cm^−1^ that can be assigned to the symmetric stretch of O–C–O, *ν*_s_(COO), and the asymmetric stretch of O–C–O, *ν*_a_(COO), respectively.^45^ Notably, in 2CuO, we have also observed peaks at 2920 cm^−1^ and 2850 cm^−1^ which can be assigned to C–H stretch, *ν*(C–H), and a combination band consisting of asymmetric O–C–O stretch and in-plane C–H bend, *ν*_comb_ = *ν*_a_(COO) + *δ*(C–H), as discussed by Hayden *et al. ν*_a_(COO) + *δ*(C–H) cannot be observed individually as they belong to the irreducible representation of *C*_2v_, which can only be attributed to the bidentate adsorption configuration of FA in the form of formate (*HCOO) on the surface.^46–48^ In 1CuO, from the absence of *ν*(C–H), the adsorption configuration can be assigned to carboxyl (*COOH) on the surface. The absence of formate binding in 1CuO can be explained by the substantial difference in the adsorption energies by our theoretical findings (as discussed later). We have further conducted electron paramagnetic resonance (EPR) experiments to verify our findings from FTIR. The EPR spectra of 1CuO and 2CuO with and without pre-adsorbed FA are presented in Fig. S33.† A characteristic Cu^2+^*g*-value of *ca.* 2.07 is obtained.^49^ It is noted that the EPR peak becomes silent in 1CuO upon the addition of FA, whereas the EPR remains active for 2CuO with FA. This indicates that the adsorption of FA on 1CuO greatly reduces the number of unpaired electrons in the system. It can be attributed to the charge transfer between Cu and C *via* the monodentate adsorption of FA on 1CuO. On the contrary, the adsorption of FA on 2CuO is bridged *via* Cu and O moieties, which could lead to a different charge transfer pathway. Clearly, the notably different EPR peaks upon FA adsorption provide highly supportive evidence on the two types of adsorption configurations.^50^ Thus, our FTIR and EPR experiments reveal that FA is adsorbed on the surface of 1CuO and 2CuO*via* monodentate (carboxyl) and bidentate mode (formate), respectively.

Subsequently, we employed DFT calculations to compare the energetic trend for both adsorption modes (see computational details in methods/ESI†). From Fig. 6b, both monodentate (carboxyl; *COOH) and bidentate (formate; *HCOO) pathways in 1CuO show similar energetically favourable trends with an overall energy release of 1.9 eV and 2.3 eV, respectively. Although the adsorption of *COOH is more favourable than *HCOO for 1CuO, the decrease in energy of the pathway *via* formate (*HCOO) is slightly larger than carboxyl (*COOH). This reveals the competition between the two pathways, which leads to a comparable product selectivity between CO and CO_2_. In contrast, the reaction trends of carboxyl and formate are notably different from 2CuO (Fig. 6c). The formate pathway displays a much more favourable energetic trend towards the formation of CO_2_, whereas the carboxyl pathway shows a low energy barrier of 0.2 eV for the conversion from *COOH to *CO. As a result, this gives a more preferred selectivity towards CO_2_ over 2CuO compared to 1CuO. Based on the structure of the active sites, adsorption energetics, and photocatalytic properties, we hereby propose two mechanisms regarding the choice in the reaction pathways. (1) 1CuO possesses only one Cu site. Therefore, FA can only be adsorbed *via* the monodentate mode. This greatly agrees with our catalytic results with a CO : CO_2_ ratio of 1.05 : 1 where the competing C–O and O–H activation pathways co-exist. (2) 2CuO and 3CuO possess multiple neighbouring Cu sites that are bridged by μ-O, which render a possible surface geometry for FA to be adsorbed in a bidentate manner that favours the dehydrogenation pathway specifically. It should be particularly noted that the monodentate adsorption mode would still be competing and inevitable (Fig. 6d).

**Fig. 7:**
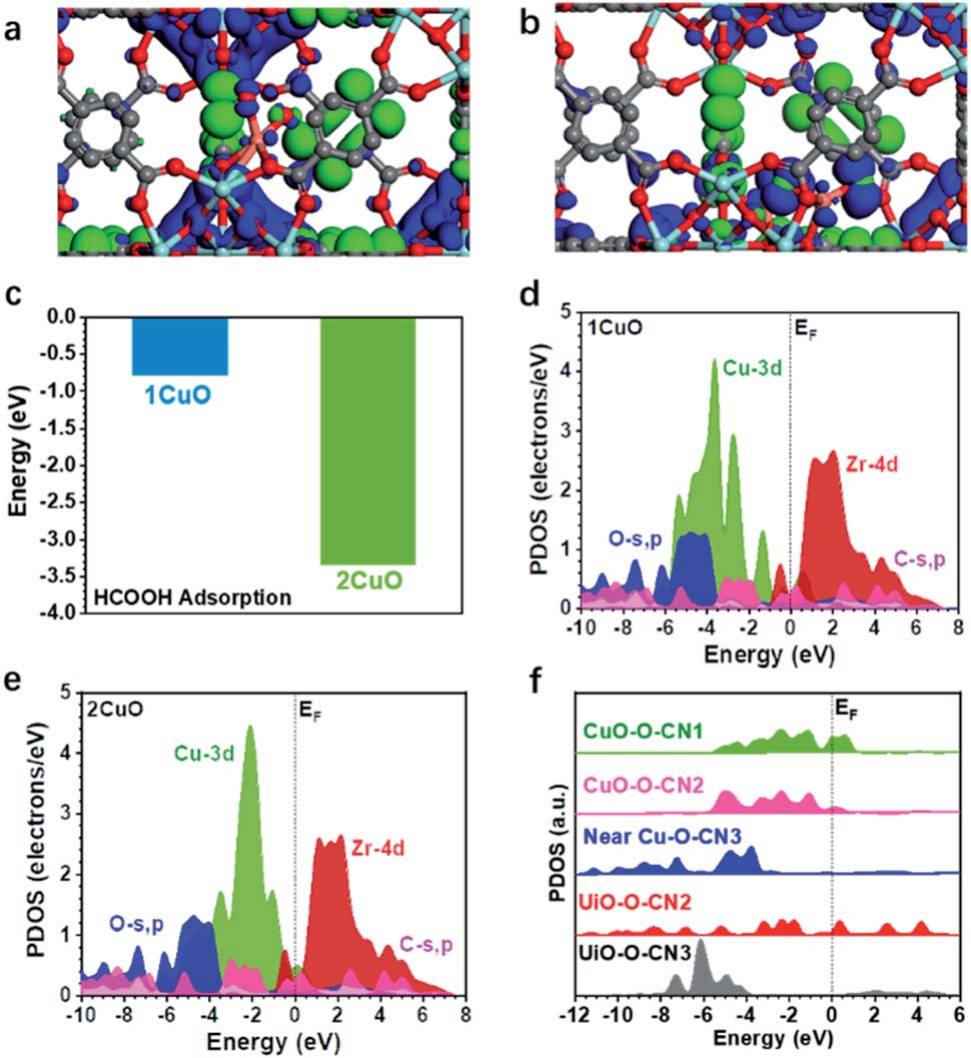
The three-dimensional contour plot of electronic distribution of (a) 1CuO and (b) 2CuO. (c) The adsorption energies of FA over 1CuO and 2CuO. The partial density of states (PDOS) of (d) 1CuO and (e) 2CuO. (f) Site-dependent PDOS of 2CuO.

The three-dimensional contour maps showing the simulated electronic structures of 1CuO and 2CuO are presented in Fig. 7a and b. The electron density of 1CuO is located near the O sites in the framework. There is a limited electronic distribution near the Cu sites, indicating a weaker electroactivity. In comparison, we notice that the electronic distribution near the Cu sites has become much more profound in 2CuO. The electronic distribution of the framework has also become slightly weaker. In both electronic structures of 1CuO and 2CuO, the aromatic (organic linker) and Zr sites mainly dominate the anti-bonding states. The introduction of the Cu sites only marginally affects the electronic structures of the host framework, which is energetically favourable for electron transfer. In addition, we have further compared the corresponding adsorption energies of FA on the active sites (Fig. 7c). The adsorption of FA is more stable on 2CuO due to a facilitated bidentate binding with the two neighbouring Cu sites, which further leads to the preferred selectivity towards CO_2_ products. Therefore, the DFT-based investigations on the electronic structures and reaction coordinates have elucidated the fundamental reason for the higher selectivity of CO_2_ over 2CuO compared to 1CuO.

We have accordingly performed more detailed investigations on the electronic structures by revealing the partial density of states (PDOS) of the structures. In 1CuO, the Cu-3d orbitals have shown a dominant peak near *E*_V_–4.0 eV (*E*_V_ denotes 0 eV) with a lower d-band centre at −3.5 eV (Fig. 7d), which indicates lower electroactivity. Meanwhile, the O-s,p orbitals are mostly located below *E*_V_–4.0 eV and the Zr-4d orbitals mainly contribute to the conduction band. In contrast, in 2CuO, the dominant peak of the Cu-3d orbitals has been significantly upshifted towards the Fermi level (*E*_F_), inferring an improved electron transfer capability in the structure (Fig. 7e).

The d-band centre of the structure has also been upshifted to −2.5 eV. Moreover, we noticed that the main structure including O-s,p, C-s,p, and Zr-4d orbitals of the host UiO-66-NH_2_ does not show any significant change in the PDOS. These results reveal that the introduction of Cu increases the electroactivity at the local regions, which promotes the FA reforming activity. As displayed in Fig. 7f, the PDOSs of O-s,p reveal the electronic structure near the Cu sites in 2CuO. Notably, the O-sites on the host framework show limited electroactivity due to the low electron density near the *E*_F_. For the O sites near Cu sites, the highly coordinated O sites (coordination number of 3) also exhibit poor electroactivity. Interestingly, we noticed that the electroactivity of O sites within the binuclear Cu_2_-oxo centre has been significantly boosted, which shows an increased electron density near *E*_F_. These electroactive sites promote FA dehydrogenation and further increase the catalytic selectivity towards CO_2_, which supports our photocatalysis results.

## Conclusions

To conclude, we have presented the controlled synthesis of Cu-oxo nanoclusters on UiO-66-NH_2_, where the nuclearity of Cu sites can be finely adjusted, by exploiting the underlying principles of coordination chemistry and solid-state chemistry. We have employed the *C*_3v_ symmetry at the ‘oxo-surface’ of the Zr_6_O_4_ nodal centre to modularly anchor different numbers of Cu sites (maximum of 3). The atomic and crystallographic parameters have been reliably determined by Rietveld refinement of SXRD, EXAFS analysis, and DFT calculations. As illustrated in the photocatalytic formic acid reforming reaction, the substantial difference between the reactive surfaces and geometric structures of the active sites leads to different adsorption modes, namely, monodentate and bidentate adsorption. This allows sophisticated control of product selectivity. A rational structure–reactivity interplay between surface chemistry, geometric structure, reaction energetics, and catalytic properties has been discerned. We believe this unique modular synthesis approach can ultimately become widely transferable to the fabrication of other well-defined nanoclusters and deserve urgent attention.

## Data availability

Data for all compounds in this manuscript are available in the ESI,† which includes general information, general procedures, experimental details, computation details, and characterisations.

## Author contributions

All authors have given approval to the final version of the manuscript.

## Conflicts of interest

There are no conflicts to declare.

## Supplementary Material

SC-013-D1SC05495C-s001
