# Supplementary material for: Controlled synthesis of Bi- and tri-nuclear Cu-oxo nanoclusters on metal–organic frameworks and the structure–reactivity correlations
Source: Chem Sci. 2021 Nov 29;13(1):50–8. doi: 10.1039/d1sc05495c (PMC8694280; doi:10.1039/d1sc05495c)
Supplement: SC-013-D1SC05495C-s001 [file SC-013-D1SC05495C-s001.pdf]

Electronic Supplementary Material (ESI) for Chemical Science.
This journal is © The Royal Society of Chemistry 2021

Supporting Information

Controlled Synthesis of bi- and trinuclear Cu-oxo Nanoclusters on Metal-organic Frameworks and the Structure-reactivity Correlations

Qi Xue, Bryan Kit Yue Ng, Ho Wing Man, Tai-Sing Wu, Yun-Liang Soo, Molly Mengjung Li, Shogo Kawaguchi, Kwok Yin Wong, Shik Chi Edman Tsang, Bolong Huang, Tsz Woon Benedict Lo**

Experimental Procedures

1. Experimental Procedures

We used a novel bottom-up approach, inspired by fundamentals of coordination chemistry, to engineer precise binuclear Cu_2-oxo and trinuclear Cu_3-oxo nanoclusters on the Zr_6O_4 nodes of UiO-66-NH_2. It is first achieved by loading a Lewis acidic Cu^{II} species onto the Lewis basic -NH_2 motif of UiO-66-NH_2 which forms a stable Lewis acid-base adduct. By utilising the di-basic chemical and geometric specificities of methylimidazolate (meIm) linkers, the 'anchored' Cu^{II} site will form a Cu-meIm complex upon the addition of meIm. This results in an open basic N-site on the coordinated imidazolate linker. A second Cu^{II} ion can hence interact with the open basic N-site, forming a binuclear Cu-complex. Extensive washing by water is needed to remove residual reaction substrates on the internal surface. Similarly, the trinuclear Cu-complex is prepared by further applying meIm and Cu^{II} precursor in a modular manner. This is similar to the concept of forming poly-amino acid structure by Fmoc solid-phase peptide synthesis. In brief, the trinuclear Cu-complex is synthesised in the following manner: -NH_2 – Cu^{II} – meIm – Cu^{II} – meIm – Cu^{II}, by utilising the underlying principles of solid-state chemistry and coordination chemistry. By calcination of the samples at 180 °C, the organic meIm linker can be removed (as shown by our TGA results). The vacant coordination sites are reactive which are hence stabilised by the neighbouring O atoms, akin to the calcination of metal-exchanged zeolites.

Synthesis of UiO-66-NH_2

The synthesis procedure is according to the previously reported UiO-66-NH_2 with the structural formula of $Zr_6O_4(OH)_4(ATA)_{5.8}$ (ATA = 2-aminoterephthalic acid).[1] 0.480 g zirconium tetrachloride and 0.372 g ATA were dissolved in 40 mL N,N-dimethylformamide (DMF). The solution was transferred into a 100 mL hydrothermal vessel and heated at 120 °C for 12 hours. The UiO-66-NH_2 powder was collected by centrifugation and washed with water 5 times. Dried at 60 °C overnight.

Synthesis of 1CuO-UiO-66-NH_2

0.47 g UiO-66-NH_2 were dispersed in 20 mL H_2O by ultrasonication. 120 μL bis(ethylenediamine)Cu^{2+} hydroxide was dropwise added. The mixture was stirred at room temperature for 12 hours. The product was collected by centrifugation and washed with 10 mL double distilled water. It was dried at 60 °C overnight to obtain the product $1Cu^{2+}$-UiO-66-NH_2. At last, the product $1Cu^{2+}$-UiO-66-NH_2 was heated to 180 °C in the oven for 2 hours to obtain 1CuO-UiO-66-NH_2. The colour of the sample changed from light lavender to wheat yellow.

Synthesis of 2CuO-UiO-66-NH_2

0.47 g UiO-66-NH_2 were dispersed in 20 mL H_2O by ultrasonication. 120 μL bis(ethylenediamine)Cu^{2+} hydroxide was dropwise added. The mixture was stirred at room temperature for 12 hours to obtain $1Cu^{2+}$-UiO-66-NH_2. The product was collected by centrifugation and washed with 10 mL double distilled water. 0.046 g 2-methylimidazole was added and stirred at room temperature for 12 hours. After centrifugation and washing the residual 2-methylimidazole with 10 mL double distilled water, 120 μL bis(ethylenediamine)Cu^{2+} hydroxide was added dropwise at room temperature and kept stirring for 12 hours to obtain $2Cu^{2+}$-UiO-66-NH_2. At last, the product was heated at 180 °C for 2 hours to yield 2CuO-UiO-66-NH_2.

Synthesis of 3CuO-UiO-66-NH_2

0.47 g UiO-66-NH_2 were dispersed in 20 mL H_2O by ultrasonication. 120 μL bis(ethylenediamine)Cu^{2+} hydroxide was dropwise added. The mixture was stirred at room temperature for 12 hours to obtain $1Cu^{2+}$-UiO-66-NH_2. The product was collected by centrifugation and washed with 10 mL double distilled water. 0.046 g 2-methylimidazole was added and stirred at room temperature for 12 hours. After centrifugation and washing the residual 2-methylimidazole with 10 mL double distilled water, 120 μL bis(ethylenediamine)Cu^{2+} hydroxide was added dropwise at room temperature and kept stirring for 12 hours to obtain $2Cu^{2+}$-UiO-66-NH_2. 0.046 g 2-methylimidazole was further added and stirred at room temperature for 12 hours. After centrifugation and washing the residual 2-methylimidazole with 10 mL double distilled water, 120 μL bis(ethylenediamine)Cu^{2+} hydroxide was also added dropwise at room temperature and kept stirring for 12 hours to obtain $3Cu^{2+}$-UiO-66-NH_2. At last, the product was heated at 180 °C for 2 hours to yield 3CuO-UiO-66-NH_2.

By calcination of the samples at 180 °C, the organic meIm linker can be removed (as shown by our TGA results). The vacant coordination sites are reactive which are hence stabilised by the neighbouring O atoms, akin to the calcination of metal-exchanged zeolites.

Formic acid (FA) reforming

5 mL 0.7 × 10^{-3} g/L catalyst was added into 5 mL 2% (v/v) FA in a quartz reactor. The reactor was then flushed with N_2 for 15 min to ensure an inert atmosphere. The photocatalytic reaction was performed using a xenon lamp (GLORIA-X500X, 500 W, 200-2500 nm, Zolix Instruments Co., Ltd.). The gaseous products were analyzed by gas chromatography (GC-2060). TCD was used for the detection of hydrogen with helium as the internal standard. FID was used for the detection of carbon monoxide and carbon dioxide.

2. Methods

High-resolution synchrotron X-ray powder diffraction (SXRD) and Rietveld refinement

High-resolution SXRD data were collected at Beamline BL02B2, SPring-8, Japan. The energy of the incident X-ray flux was set at 18 keV. The tuned energy for each beamline emits the optimum X-ray flux to achieve high contrast (signal-to-noise ratio) and high angular resolution. The wavelength and the 2θ-zero point were calibrated using a diffraction pattern obtained from a high-quality standard CeO_2 powder. High-resolution SXRD data were obtained from the zeolite samples (loaded in 0.5-mm borosilicate capillaries) using the MYTHEN detector. Each diffraction pattern was collected for an hour for good statistics. In total, there are 490 *hkl* reflections measured (within the region of refinement (2θ = 2-60°), of which at least 100 independent *hkl* reflections are observed. From a mathematical perspective, the number of variables should not exceed the number of observables. In the Rietveld refinements performed in this work, the number of structural parameters has not exceeded 40.

Using the TOPAS 6.0 software, the lattice parameters were obtained using Pawley and Rietveld refinement analyses of the diffraction patterns were performed. The background curve was fitted by a Chebyshev polynomial with an average of 16 coefficients. The Thompson-Cox-Hastings (pseudo-Voigt) function was applied to describe the diffraction peaks[2]. The scale factor and lattice parameters were allowed to vary for all the histograms.

The final refined structural parameters for each data histogram were carried out using the Rietveld method with the fractional coordinates (*x, y, z*) and isotropic displacement factors (B_{eq}) for all atoms. In addition, the R_{wp} and *goodness-of-fit* values ($gof = R_{wp}/R_{exp}$) are helpful to indicate the quality of fit, where R_{exp} represents the quality of the data.

(i) Framework atoms

The crystallographic locations of the framework atoms may change slightly – a small deviation from the model by Wragg *et al*. Therefore, before the refinement of the entire structure with the guest Cu site, the framework atoms and the positions of atoms were first refined. This was performed to avoid a miscalculation of the structure that reaches the global minimum of the refinement by changing the entire framework.

(ii) Fourier analysis

The Fourier analysis was used to identify the positions with the highest remaining electron density in the framework, once the positions of the host atoms have been determined.

(iii) Inclusion of guest molecules

Based on the Fourier analysis, a Monte Carlo-based simulated annealing technique in which the guest Cu complex as the rigid body was used to locate their positions in the UiO-66-NH_2. The guest Cu complex rigid body Z-matrices were refined while keeping the fractional coordinates of the framework atoms fixed.

It was first applied to be simulated annealed using the Rietveld method (while the fractional coordinates of the framework were fixed). Thus, the simulated annealing technique ensures the correct number of Cu complexes.

After identifying the number and location of the Cu sites, the site occupancy factors (SOFs) were refined. Also, the bond and dihedral angles of Cu-OH in the rigid-body were refined with restraining to ± 20% from the optimized tetrahedral structures using Chem3D software. Then, the relevant parameters were relaxed to be refined by simulated annealing repeatedly for an hour to ensure the global minimum has been reached. The global minimum is indicated by the lowest R_{wp} and *gof* values.

Several criteria were met to ensure the high quality and reliability of the refinement, namely, (i) the global minimum has been reached, (ii) the derived crystal structure fits chemical sense, (iii) reasonable systematic error values for all the refined parameters, and (iv) sensible SOF and B_{eq} values.

The B_{eq} were constrained in the following way: (i) all the framework Zr-sites share the same value as 0.6 Å^2, and the values for the framework O-sites as 2 Å^2, and (ii) the B_{eq} of the extra-framework atoms were all arbitrary fixed at 8-12 Å^2 (as B_{eq} is broadly accepted to be about proportional to measurement temperature as that of framework sites)[3–7]. The position errors of the Cu complex were estimated from the percentage errors of the translation and rotation axes of the rigid bodies.

High-throughput SXRD

High-throughput SXRD measurements for in situ desorption study were collected at beamline BL02B2 at SPring-8, Japan. The energy of the incident X-ray flux was set at 18 keV[2]. The wavelength (λ = 0.689556(2) Å) and the 2θ zero-point (ZP = -0.000015(2)°) were calibrated using a diffraction pattern obtained from a high-quality CeO_2 powder (NIST SRM674b). High-throughput SXRD data were obtained from the zeolite samples (loaded in 0.5-mm borosilicate capillaries)) using the MYTHEN detector. The patterns were collected in the 2θ range 2-78° with 0.006° data binning. Each SXRD pattern was collected for 5 min for each MYTHEN-2θ-step, *i.e.*, 10 min in total for MYTHEN data summation. This produced patterns with a good signal-to-noise ratio (S/N). Therefore, the quality of the Rietveld refinement should be best judged by the difference between the fitted and observed data.

In-situ PXRD measurements

Pre-treated samples were finely sieved and loaded in 0.5-mm-borosilicate capillaries to reduce the X-ray absorption problem. High-energy X-ray using Mo anode was used to optimize the spatial and angular resolution of Bragg's reflections. Dynamic measurements were conducted at elevated temperatures at 5 °C min^{-1}. Each powder XRD pattern required 15 min of scanning time for a suitable and reliable signal-to-noise ratio.

Extended X-ray absorption fine structure spectroscopy

The extended X-ray absorption fine structure (EXAFS) spectroscopy data were collected at BL07A at Taiwan Light Source using transmission mode, with an average scanning time of 20 minutes. Artemis and Athena software were used for data treatment and analysis.[8] The detailed fitting parameters are summarized in the caption of the EXAFS fittings. The Hamma software was used for wavelet transform[9].

Fourier-transform Infrared (FTIR) spectroscopy

FTIR spectroscopy experiments were performed in Thermo Scientific Nicolet IS50 with attenuated total reflection (ATR) mode. The samples (10 mg, 1CuO and 2CuO) were treated by adsorbing 30 μL FA. The samples without FA adsorption were used as backgrounds. The spectra of adsorbed FA on 1CuO and 2CuO were collected by subtracting the corresponding backgrounds.

Calculation Setup for $1Cu^{2+}$- $2Cu^{2+}$-, $3Cu^{2+}$, and $4Cu^{2+}$-UiO-66-NH_2

For all the calculations within this work, we have applied the DFT calculations within the CASTEP code.[10] The GGA and PBE exchange-correlation functionals are selected for all the calculations.[11,12] The cutoff energy of plane-wave basis sets based on the ultrasoft pseudopotential has been set to 440 eV with the selection of the algorithm Broyden-Fletcher-Goldfarb-Shannon (BFGS) for all the geometry optimizations.[13] The Monkhost-Pack reciprocal space integration was performed using coarse k-points with a mesh of 2×2×1[14], which was guided by the initial convergence test. With these settings, the overall total energy for each step is converged to less than 5.0×10^{-5} eV per atom. The Hellmann-Feynman forces on the atom were converged to less than 0.001 eV/Å.

Calculation Setup for 1CuO-, 2CuO, and 3CuO-UiO-66-NH_2

We have applied the DFT calculations based on the CASTEP packages to investigate the electronic structures and energetic trend of formic acid decomposition.[10] To accurately describe the exchange-correlation energy, we have selected the generalized gradient approximation (GGA) and Perdew-Burke-Ernzerhof (PBE) for this work.[11,12,15] The cutoff energy of the plane-wave basis has been set to 440 eV with the ultrafine quality with the utilization of the ultrasoft pseudopotentials for all the geometry optimizations. The Broyden-Fletcher-Goldfarb-Shannon (BFGS) algorithm has been applied in this work with the coarse quality of k-points for all the energy minimizations in this work.[13] To accomplish the geometry optimizations, the convergence test requires the total energy difference should be less than 5×10^{-5} eV per inter-ionic displacement as 0.005 Å per atom, respectively.

Results and Discussion

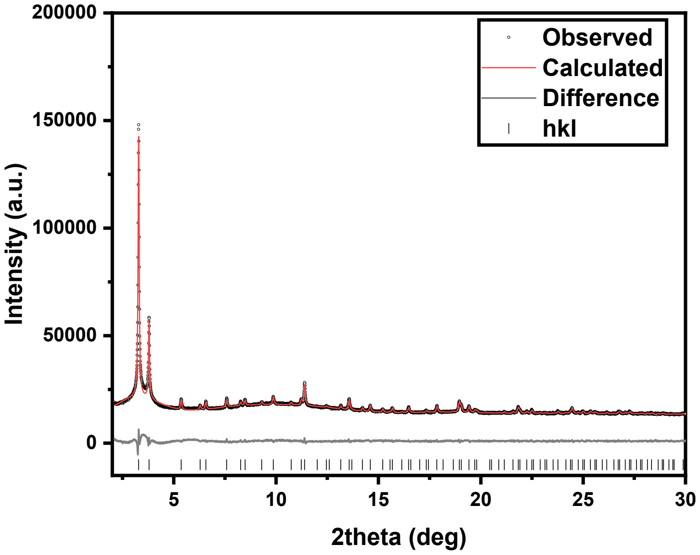

Figure S1. SXRD pattern of UiO-66-NH_2. Data collected in Beamline BL02B2 in SPring-8 (E = 18 keV). Ultra-high crystallinity can be observed.

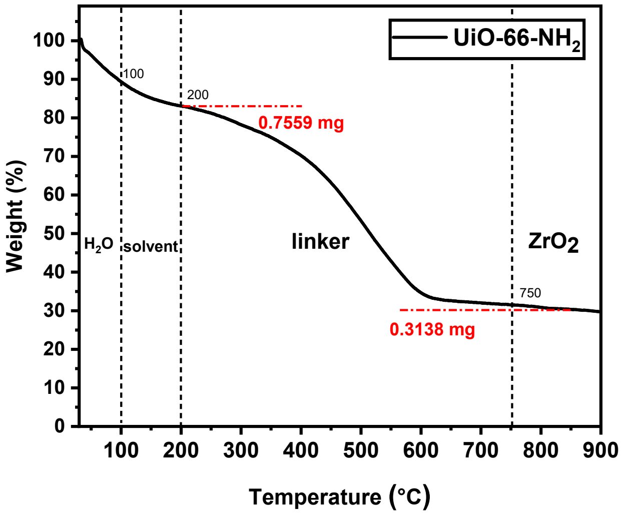

Figure S2. Thermogravimetric analysis of pristine UiO-66-NH_2 for the extrapolation of linker defect.

To calculate the number of moles of ATA organic linker:

$$n(ATA) = \frac{m}{M} = \frac{(0.7559 - 0.3138)mg}{181.14\ g/mol} = 2.44\ \times 10^{-3}\ mmol$$

To calculate the number of moles of ZrO_2:

$$n(ZrO_2) = \frac{m}{M} = \frac{0.3138\ mg}{123.22\ g/mol} = 2.55\ \times 10^{-3}\ mmol$$

Thus, the ratio of Zr:ATA = 1:0.957. The chemical formula is, therefore, $Zr_6O_4(OH)_4(ATA)_{5.7}$, where ATA = 2-aminoterephthalate.

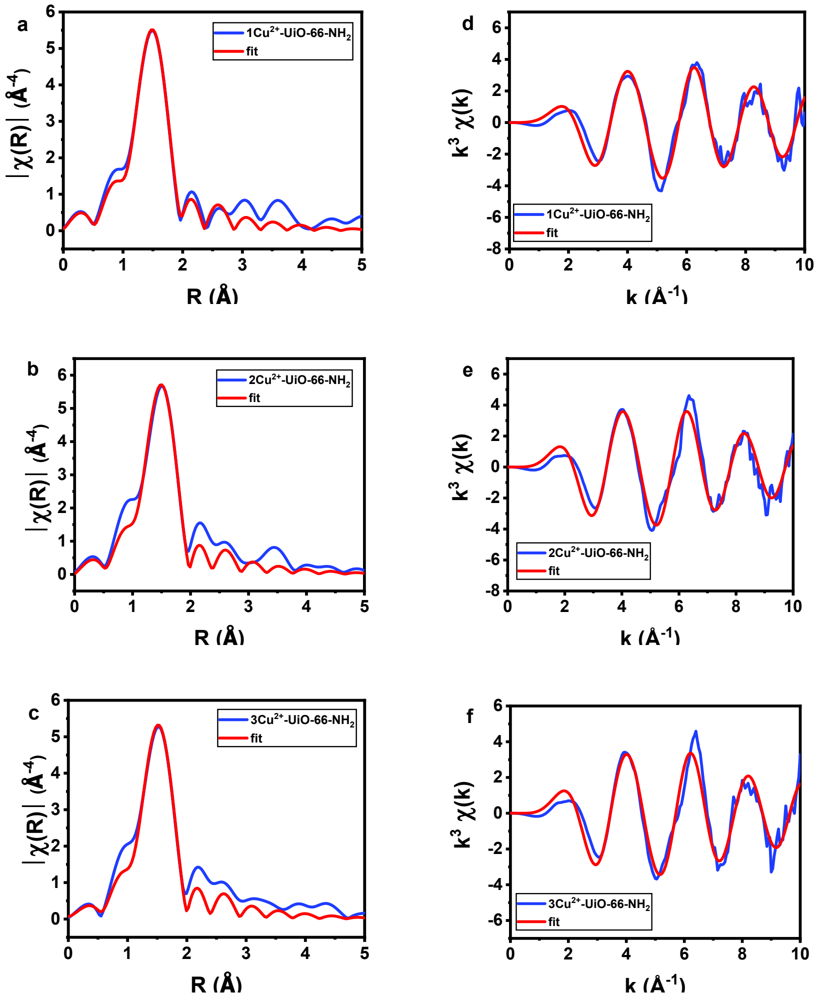

Figure S3. Cu K-edge EXAFS (blue) and fitting (red) for (a) $1Cu^{2+}$-UiO-66-NH_2, (b) $2Cu^{2+}$-UiO-66-NH_2, and (c) $3Cu^{2+}$-UiO-66-NH_2 shown in k^3 weighted *R*-space. (d-f) The corresponding plots of the *k*-spaces.

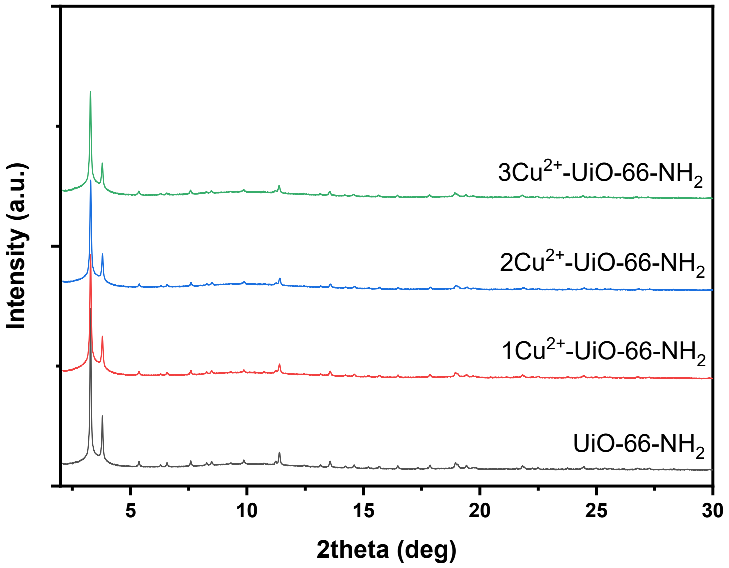

Figure S4. SXRD patterns of UiO-66-NH_2, 1Cu^{2+}-UiO-66-NH_2, 2Cu^{2+}-UiO-66-NH_2, and 3Cu^{2+}-UiO-66-NH_2. The crystal structure of UiO-66-NH_2 is preserved. No new crystalline peak is found, indicative of the absence of new phases, *e.g.*, Cu or CuO.

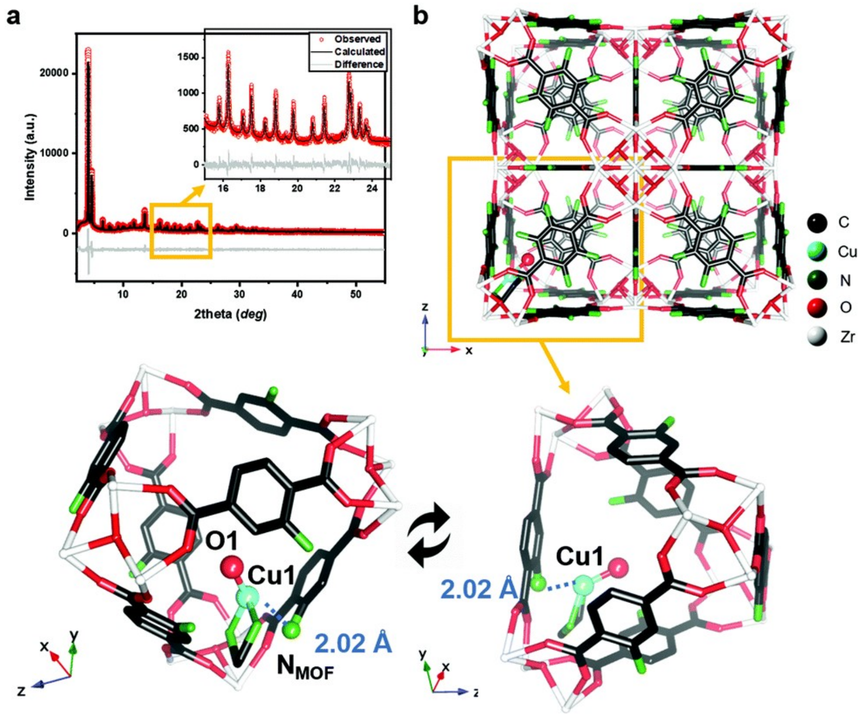

Figure S5. Rietveld refinement of SXRD data of (a) 1Cu^{2+}-UiO-66-NH_2 and (b) the corresponding refined crystal structure. Pseudo-tetrahedral geometry of the Cu^{2+} site has been reported in our recent work.[1] A zoom-in image of an asymmetric unit is shown.

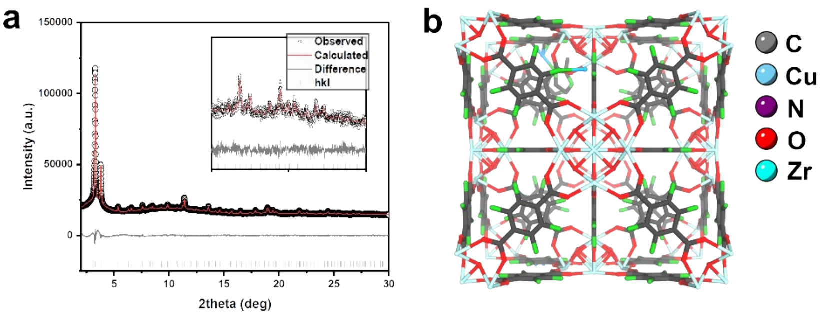

Figure S6. Rietveld refinement of SXRD data of (a) 2Cu^{2+}-UiO-66-NH_2 and (b) the corresponding refined crystal structure. A zoom-in image of an asymmetric unit is shown.

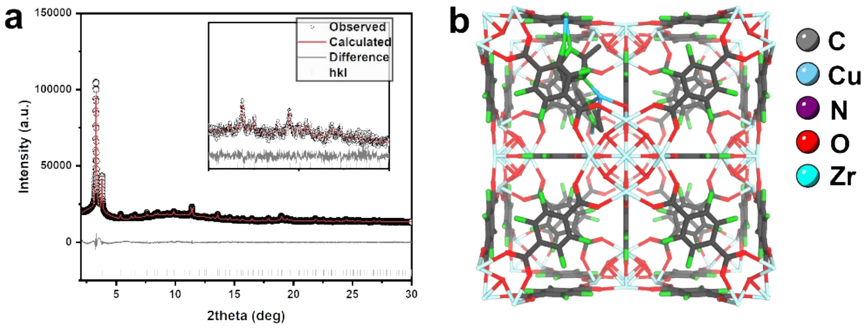

Figure S7. Rietveld refinement of SXRD data of (a) $3Cu^{2+}$-UiO-66-NH_2 and (b) the corresponding refined crystal structure. A zoom-in image of an asymmetric unit is shown.

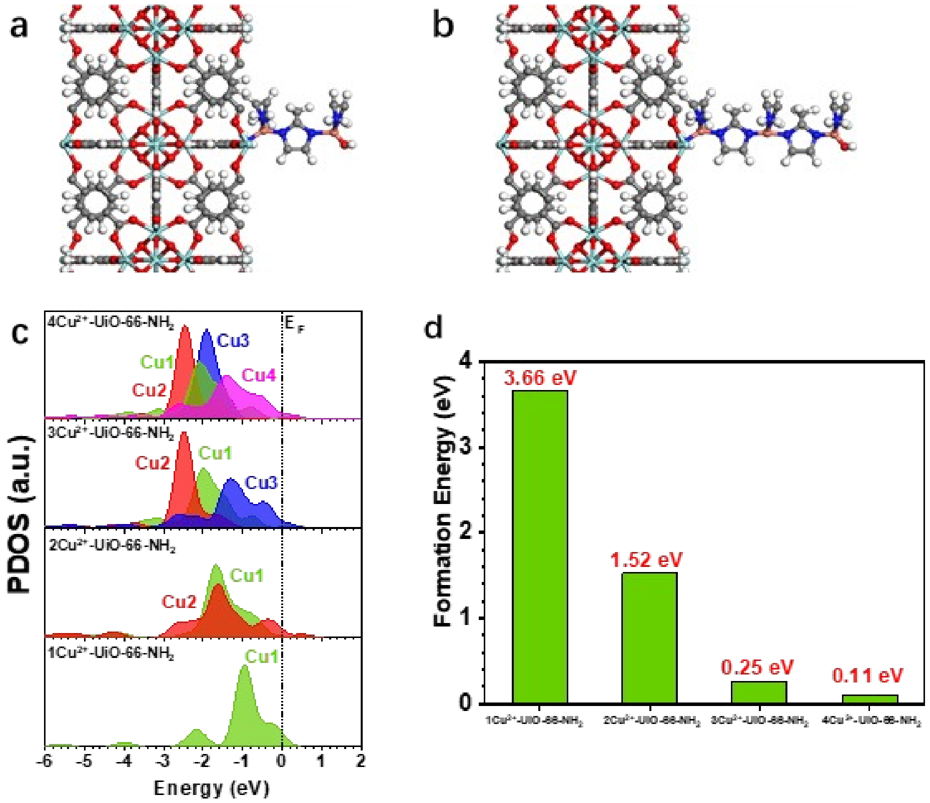

Figure S8. The relaxed structure of (a) $2Cu^{2+}$-UiO-66-NH_2 and (b) $3Cu^{2+}$-UiO-66-NH_2. Zr (cyan), Cu (orange), N (blue), O (red), C (grey) and H (white). (c) The PDOSs comparison of Cu-3d orbitals in $1Cu^{2+}$- to $4Cu^{2+}$-UiO-66-NH_2. (d) The calculated formation energies of $1Cu^{2+}$-UiO-66-NH_2 to $4Cu^{2+}$-UiO-66-NH_2.

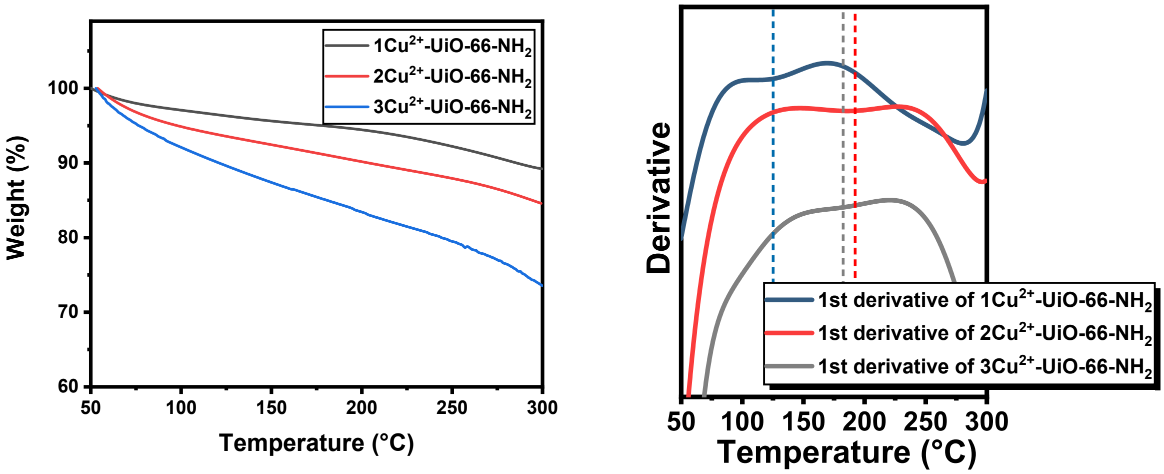

Figure S9. Thermogravimetric analyses of $1Cu^{2+}$-UiO-66-NH_2, $2Cu^{2+}$-UiO-66-NH_2, and $3Cu^{2+}$-UiO-66-NH_2. Inset: derivation of weight loss from 50 °C to 300 °C.

Calculation steps for the extrapolation of Cu:meIm ratios in the samples.

Mass (g)	@ 100 °C	@ 180 °C	Difference
$1Cu^{2+}$-UiO-66-NH_2	0.9276	0.9077	0.0199
$2Cu^{2+}$-UiO-66-NH_2	0.9538	0.9109	0.0429
$3Cu^{2+}$-UiO-66-NH_2	0.9746	0.9066	0.0680

Molar mass of a unit cell of UiO-66-NH_2: 6848.10 g/mol ($Zr_{24}O_{120}C_{192}H_{96}N_{24}$)

Mass of water and solvent to be determined using $1Cu^{2+}$-UiO-66-NH_2: 0.0199 g

Molar mass of $2Cu^{2+}$-UiO-66-NH_2
= 6848.10 + 5.04 Cu (from elemental analysis) x (63.5 + 60) = 7470.5 g/mol
No. of mole of Cu in $2Cu^{2+}$-UiO-66-NH_2 = 5.04 x 0.9109/7470.5 = 6.12 x 10^{-4}
No. of mole of meIm in $2Cu^{2+}$-UiO-66-NH_2 = (0.0429 - 0.0199)/82.1 = 2.80 x 10^{-4}
Ratio of Cu:meIm = **2.18:1**

Molar mass of $3Cu^{2+}$-UiO-66-NH_2
= 6848.10 + 7.20 Cu (from elemental analysis) x (63.5 + 60) = 7737.3 g/mol
No. of mole of Cu in $3Cu^{2+}$-UiO-66-NH_2 = 7.2 x 0.9066/7737.3 = 8.44 x 10^{-4}
No. of mole of meIm in $2Cu^{2+}$-UiO-66-NH_2 = (0.0680 - 0.0199)/82.1 = 5.86 x 10^{-4}
Ratio of Cu:meIm = **1.44:1**

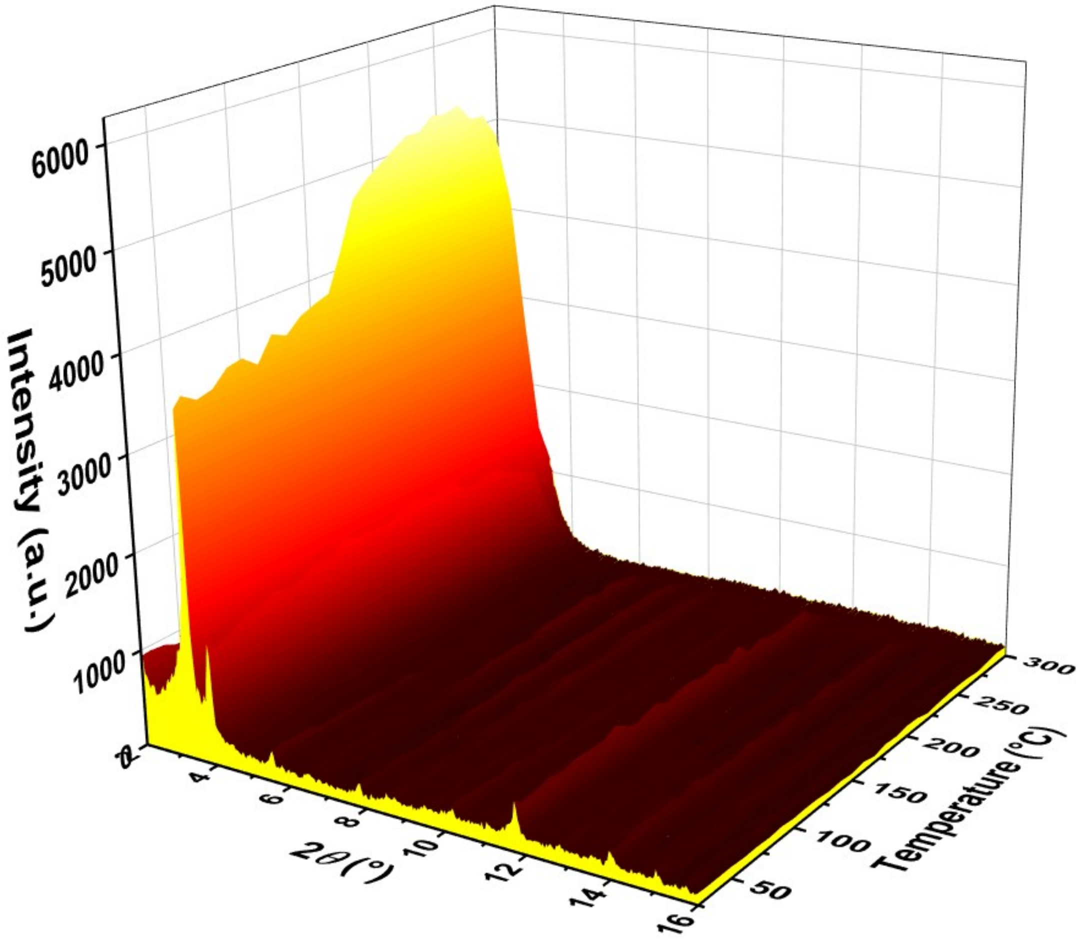

Figure S10(a). In-situ XRD of $1Cu^{2+}$-UiO-66-NH_2 from 25 °C to 300 °C.

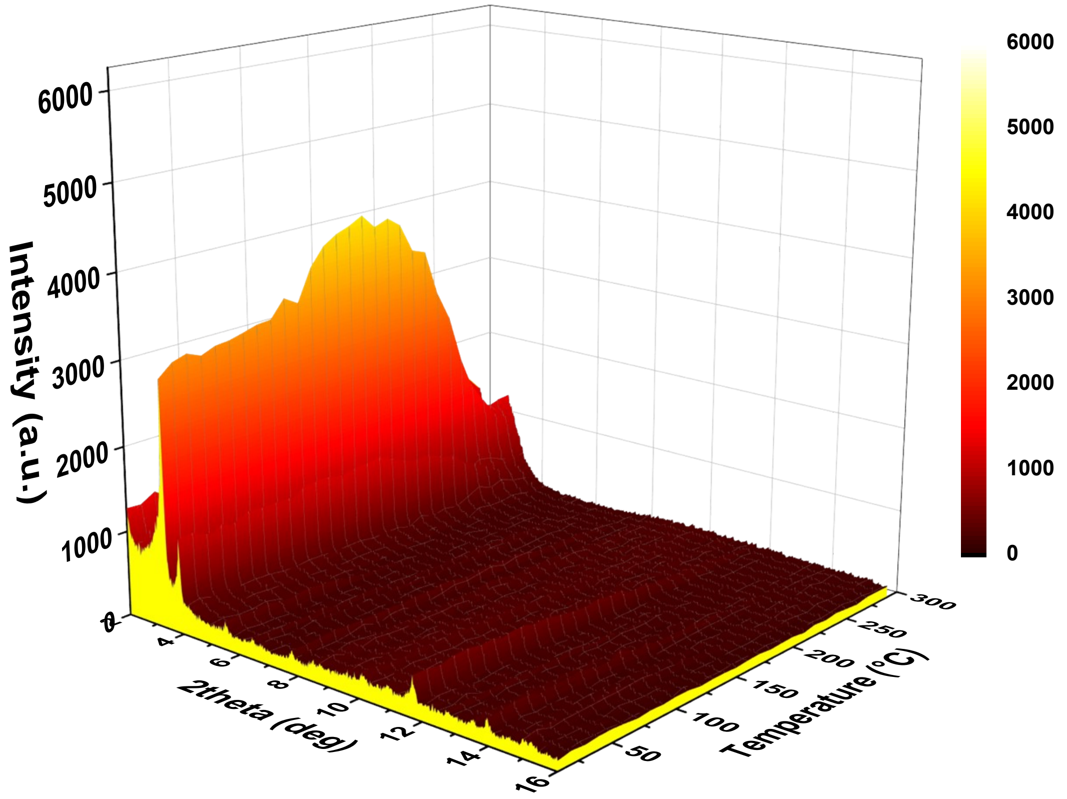

Figure S10(b). In-situ XRD of $2Cu^{2+}$-UiO-66-NH_2 from 25 °C to 300 °C.

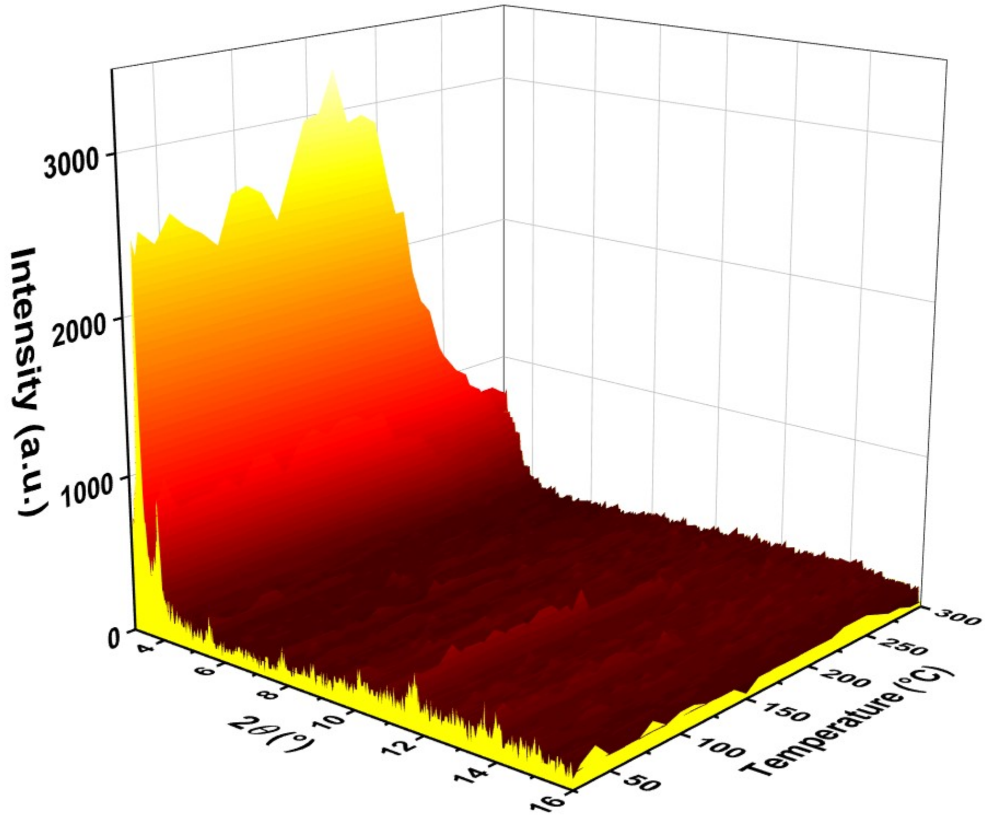

Figure S10(c). In-situ XRD of $3Cu^{2+}$-UiO-66-NH_2 from 25 °C to 300 °C.

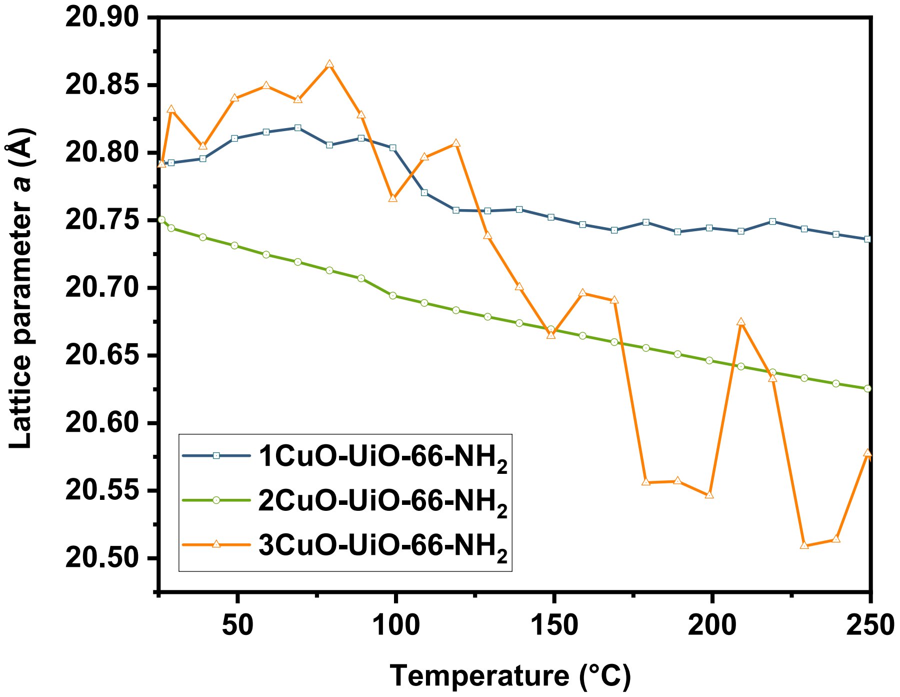

Figure S11. The dynamic change of the refined lattice parameters *a* derived from *in-situ* PXRD by Pawley refinement using TOPAS v.6.0.

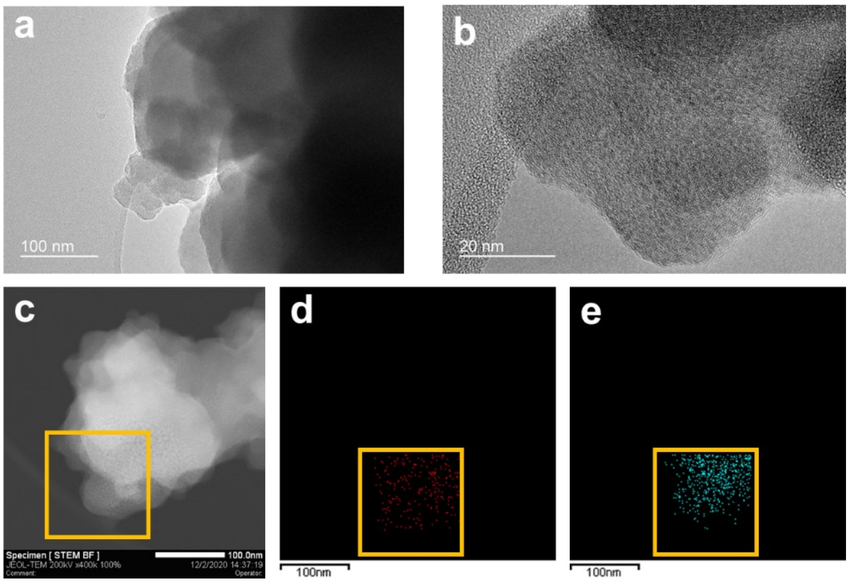

Figure S12. Scanning transmission electron micrographs of 1CuO-UiO-66-NH_2 with the scale bar of (a) 100 nm and (b) 20 nm. (c) The EDX mapping area. The distributions of Cu and Zr are shown in (d) and (e).

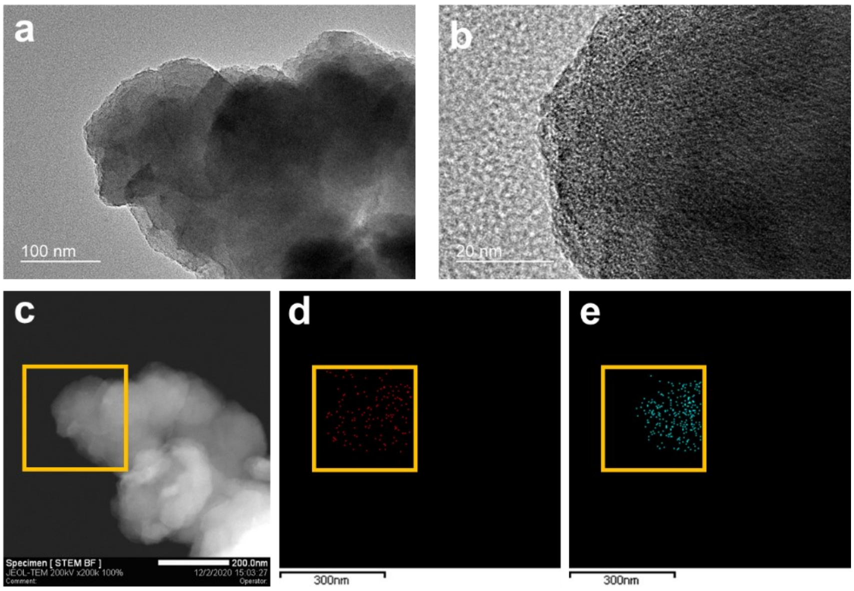

Figure S13. Scanning transmission electron micrographs of 2CuO-UiO-66-NH_2 with the scale bar of (a) 100 nm and (b) 20 nm. (c) The EDX mapping area. The distributions of Cu and Zr are shown in (d) and (e).

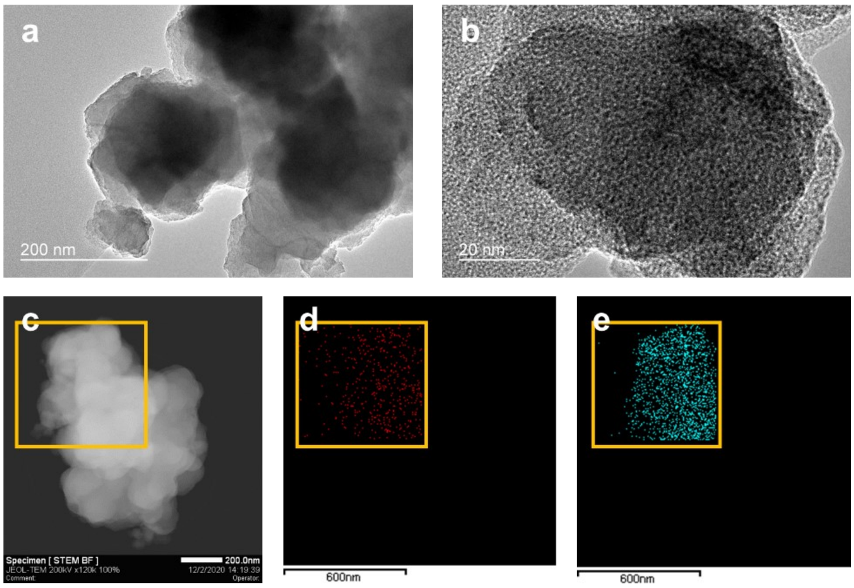

Figure S14. Scanning transmission electron micrographs of 3CuO-UiO-66-NH_2 with the scale bar of (a) 200 nm and (b) 20 nm. (c) The EDX mapping area. The distributions of Cu and Zr are shown in (d) and (e).

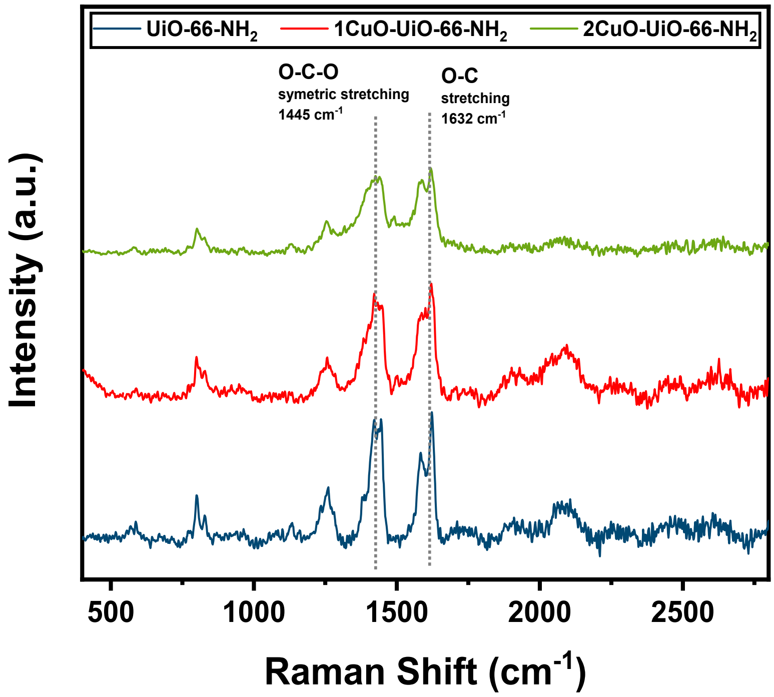

Figure S15. Raman spectra of UiO-66-NH_2, 1CuO-UiO-66-NH_2 and 2CuO-UiO-66-NH_2. The parent structure of UiO-66-NH_2 remains upon metalation.

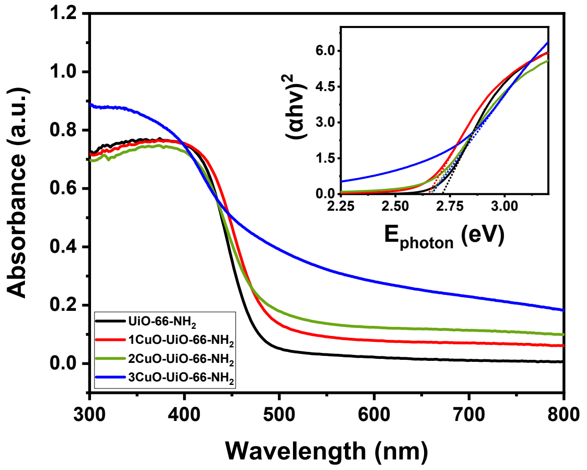

Figure S16. UV-vis spectra showing the reflectance across the UV visible wavelengths. Inset: Tauc plots were used to estimate the optical band gaps. A slight decrease can be observed in 1CuO-UiO-66-NH_2, 2CuO-UiO-66-NH_2, and 3CuO-UiO-66-NH_2 compared to UiO-66-NH_2.

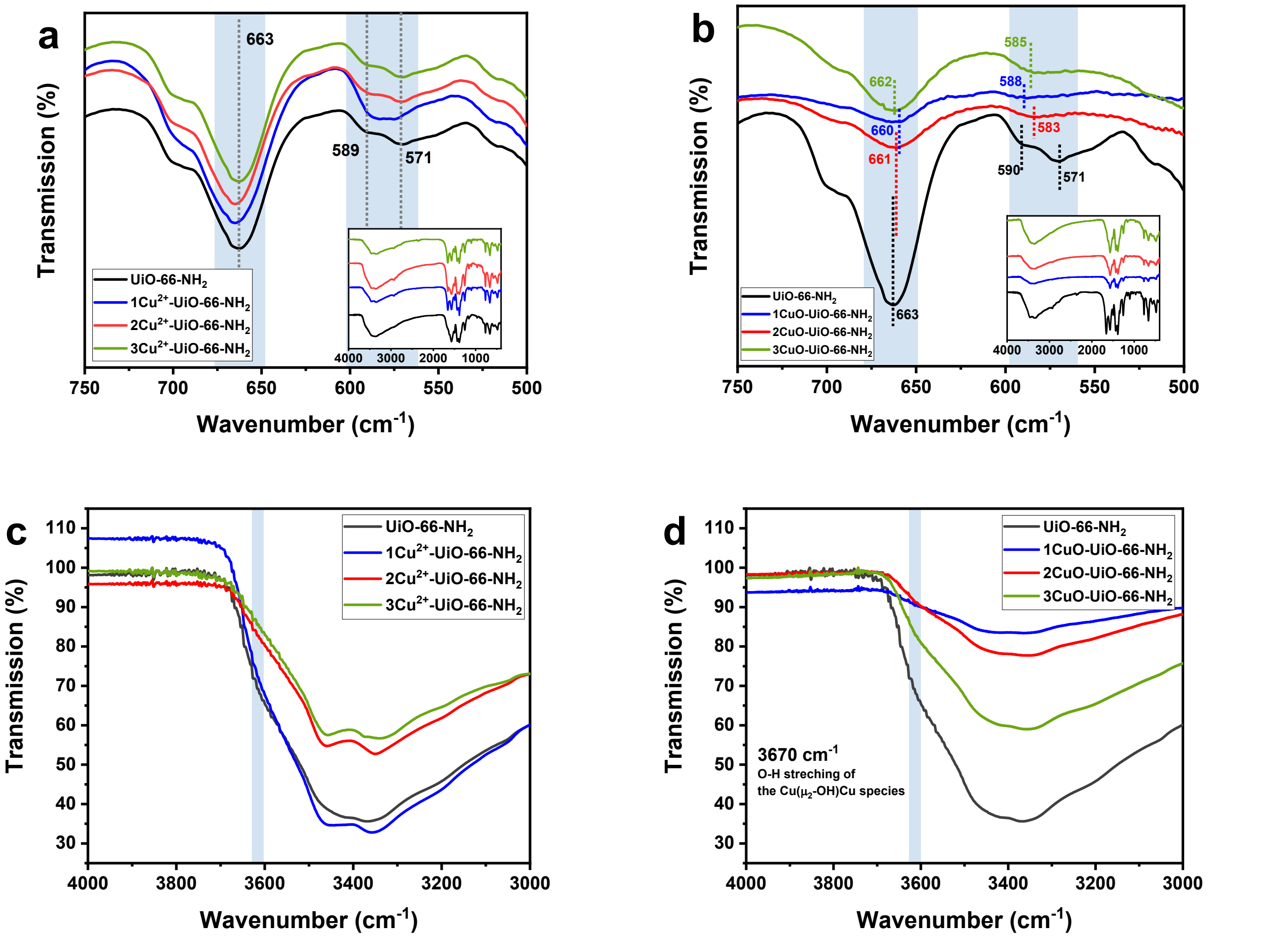

Figure S17. FTIR spectra of (a) UiO-66-NH_2, $1Cu^{2+}$-UiO-66-NH_2, $2Cu^{2+}$-UiO-66-NH_2 and $3Cu^{2+}$-UiO-66-NH_2 in the range from 750 cm^{-1} to 500 cm^{-1}. Inset: The FTIR spectra in the range from 4000 cm^{-1} to 400 cm^{-1}. FTIR spectra of (b) UiO-66-NH_2, 1CuO-UiO-66-NH_2, 2CuO-UiO-66-NH_2, and 3CuO-UiO-66-NH_2 in the range from 750 cm^{-1} to 500 cm^{-1}. Inset: The FTIR spectra in the range from 4000 cm^{-1} to 400 cm^{-1}. The change of the peak intensity and positions were ascribed to the interaction between Cu and Zr-O metal nodes. (c) $1Cu^{2+}$-UiO-66-NH_2, $2Cu^{2+}$-UiO-66-NH_2 and $3Cu^{2+}$-UiO-66-NH_2 in the range from 4000 cm^{-1} to 3000 cm^{-1}. (d) 1CuO-UiO-66-NH_2, 2CuO-UiO-66-NH_2, and 3CuO-UiO-66-NH_2 in the range from 4000 cm^{-1} to 3000 cm^{-1}. A broad peak of O-H stretching of the Cu(μ_2-OH)Cu species at around 3670 cm^{-1} can be observed after calcination.

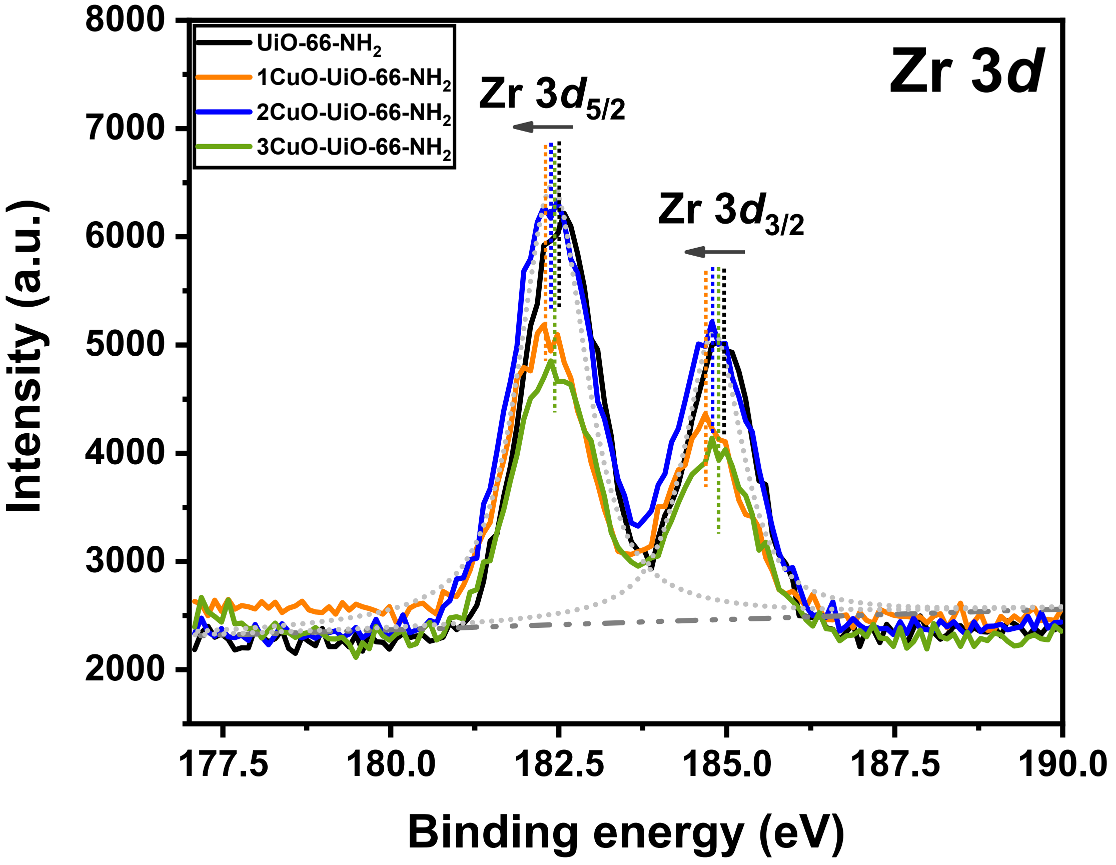

Figure S18. X-ray photoelectron spectra of Zr 3*d* range for UiO-66-NH_2 (black), 1CuO-UiO-66-NH_2 (orange), 2CuO-UiO-66-NH_2 (blue), and 3CuO-UiO-66-NH_2 (green).

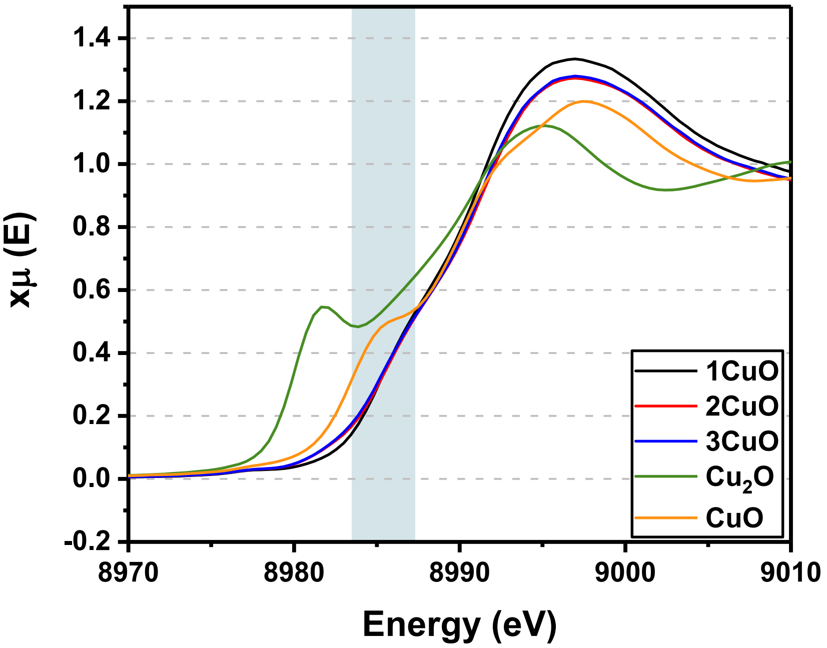

Figure S19. X-ray absorption near-edge structures of 1CuO-UiO-66-NH_2, 2CuO-UiO-66-NH_2, and 3CuO-UiO-66-NH_2, and reference CuO and Cu_2O.

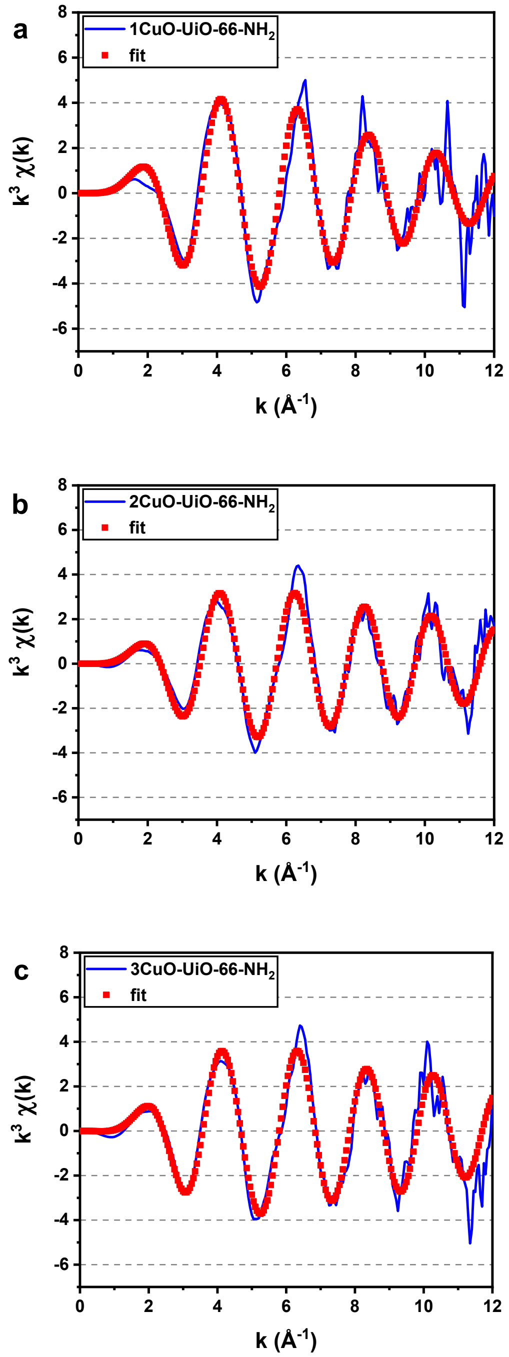

Figure S20. Cu K-edge EXAFS (line) and fitting (points) for (a) 1CuO-, (b) 2CuO-, and (c) 3CuO-UiO-66-NH_2 shown in k^3 weighted *k*-space.

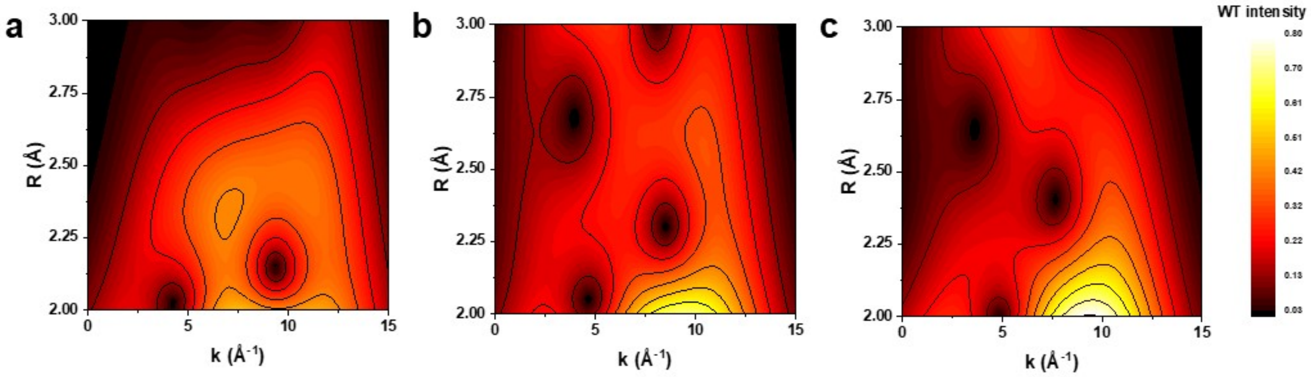

Figure S21. Wavelet transforms for the EXAFS signals of (a) 1CuO-, (b) 2CuO-, and (c) 3CuO-UiO-66-NH_2 in the R range between 2.00 and 3.00 Å.

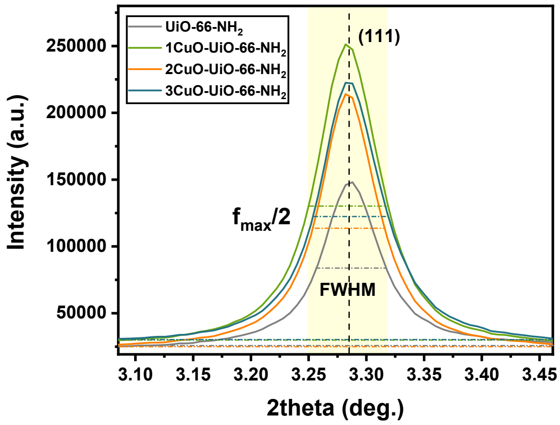

Figure S22. Comparison of the Bragg peak (111) of UiO-66-NH_2, **1CuO**, **2CuO** and **3CuO**. Data was collected on Beamline BL02B2 at SPring-8.

Investigation on whether the Cu sites are homogeneously dispersed inside MOFs. Typically, these structural parameters can be obtained from the quantitative analysis of the diffraction measurements. It should also be noted that we have particularly chosen to perform the SXRD measurements on BL02B2 at SPring-8 because of the highly optimised optical and instrumental configurations for the analysis of homo/heterogeneity.[16]

The (111) reflection (2theta = 3.27°) as an example, peak patterns of the UiO-66-NH_2, 1CuO, 2CuO, and 3CuO samples are compared in Figure S24. Theoretically, if the extra-framework species were disordered inside MOFs (such as more metals closer to the surface of the MOF), incoherent diffraction would be generated. As a consequence, the peak position will be partially moved, leading to symmetric peaks. Also, the Bragg peaks of **1CuO**, **2CuO**, and **3CuO** are all symmetrical (see Table S6; all at 0.0001), indicating the samples are very homogeneous. This suggests that the metalation processes (in **1CuO**, **2CuO**, and **3CuO**) are also homogeneous, which agrees with our recent study on the single-atom metalation on UiO-66-NH_2.[1]

By further quantitative analysis of the SXRD patterns (**Table S6**), we have obtained the volume-weighted mean column heights, LVol which are calculated using FWHMs and integral breadths (IB) assuming intermediate crystallite size broadening modelled by Voigt function. Similarly, the micro-strain, e_0 (dislocations, vacancies, and other defects) was calculated from FWHMs. We found the symmetry of UiO-66-NH_2 (peak asymmetry parameter of 0.0001) remains after the fabrication of **1CuO**, **2CuO**, and **3CuO**, which confirms the homogeneous and ordered dispersion of Cu sites inside MOFs.

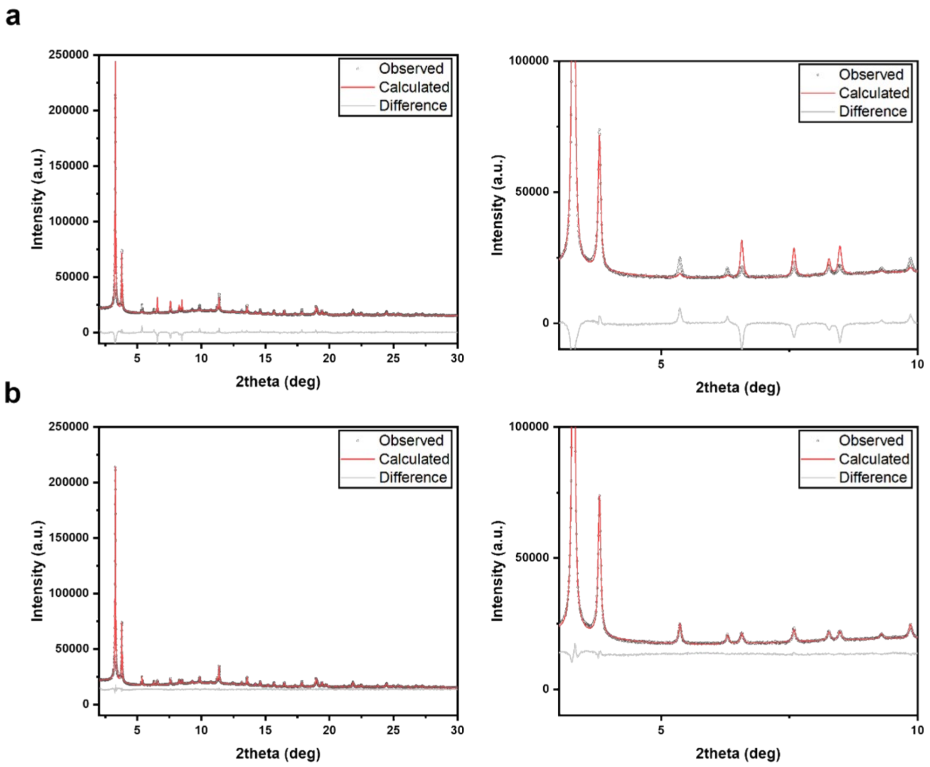

Figure S23. The Rietveld refinement fitting profiles of **1CuO** (a) without the extra-framework 'Cu-oxo' species, and (b) with the extra-framework 'Cu-oxo' species.

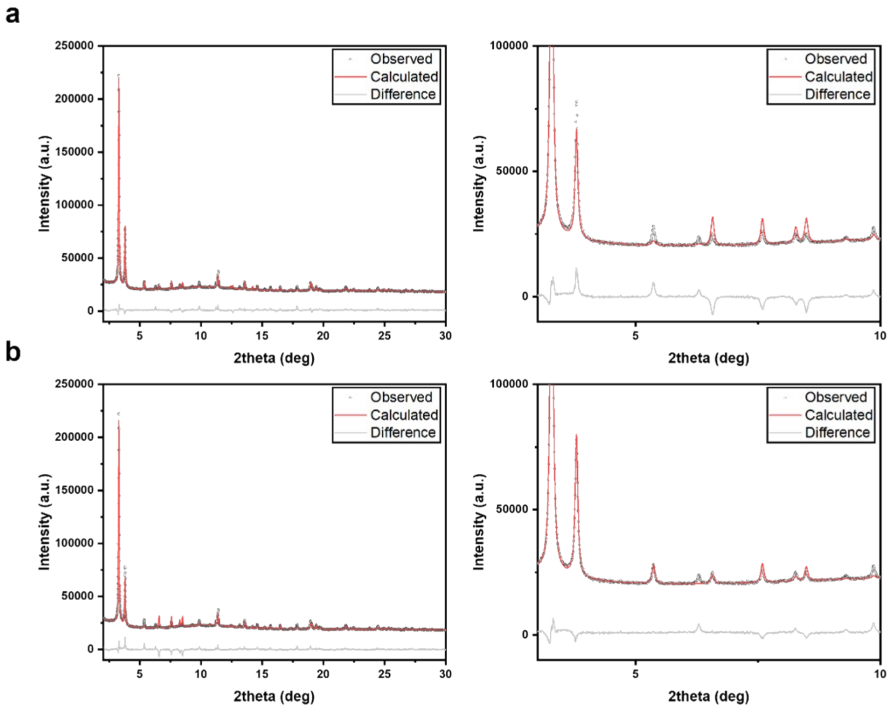

Figure S24. The Rietveld refinement fitting profiles of **2CuO** (a) without the extra-framework 'Cu-oxo' species, and (b) with the extra-framework 'Cu-oxo' species.

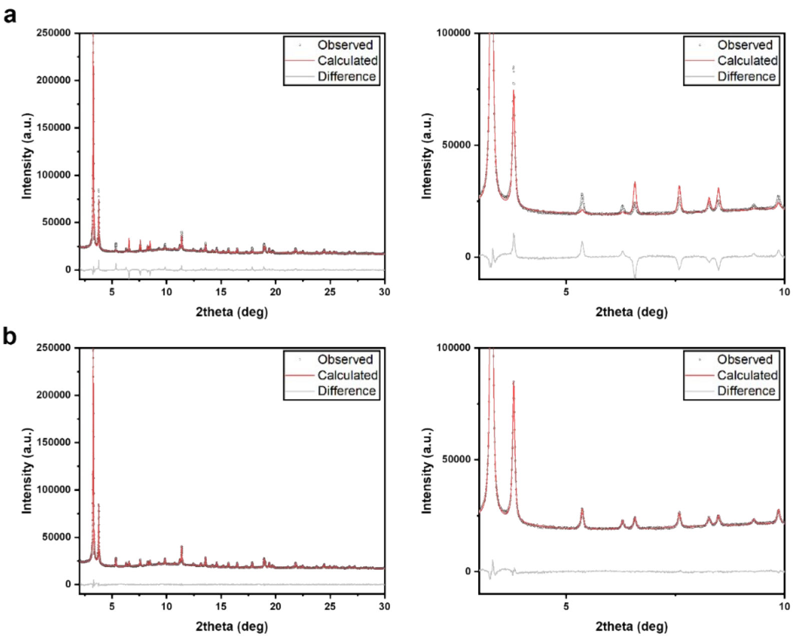

Figure S25. The Rietveld refinement fitting profiles of **3CuO** (a) without the extra-framework ‘Cu-oxo’ species, and (b) with the extra-framework ‘Cu-oxo’ species.

Cu-modified UiO-66-NH_2 samples with and without the Cu-oxo species to improve the reliability and confidence of the fittings. As presented in Figures S23-25 and Table S7, the impact on the removal of the Cu-oxo species from the refinement is substantial where the mismatch in fitting is particularly obviously between 2θ = 5-10°, which primarily have been ascribed to the occupation of the extra-framework species.[17] Furthermore, we have also systematically presented a comparison between the Bragg peak intensities of **1CuO**, **2CuO**, and **3CuO** in **Table S8**, as it is difficult to virtually see the differences in a standard graphical presentation. Clearly, obvious differences in the peak intensities can be seen in reflections such as (222), (331), and (440).

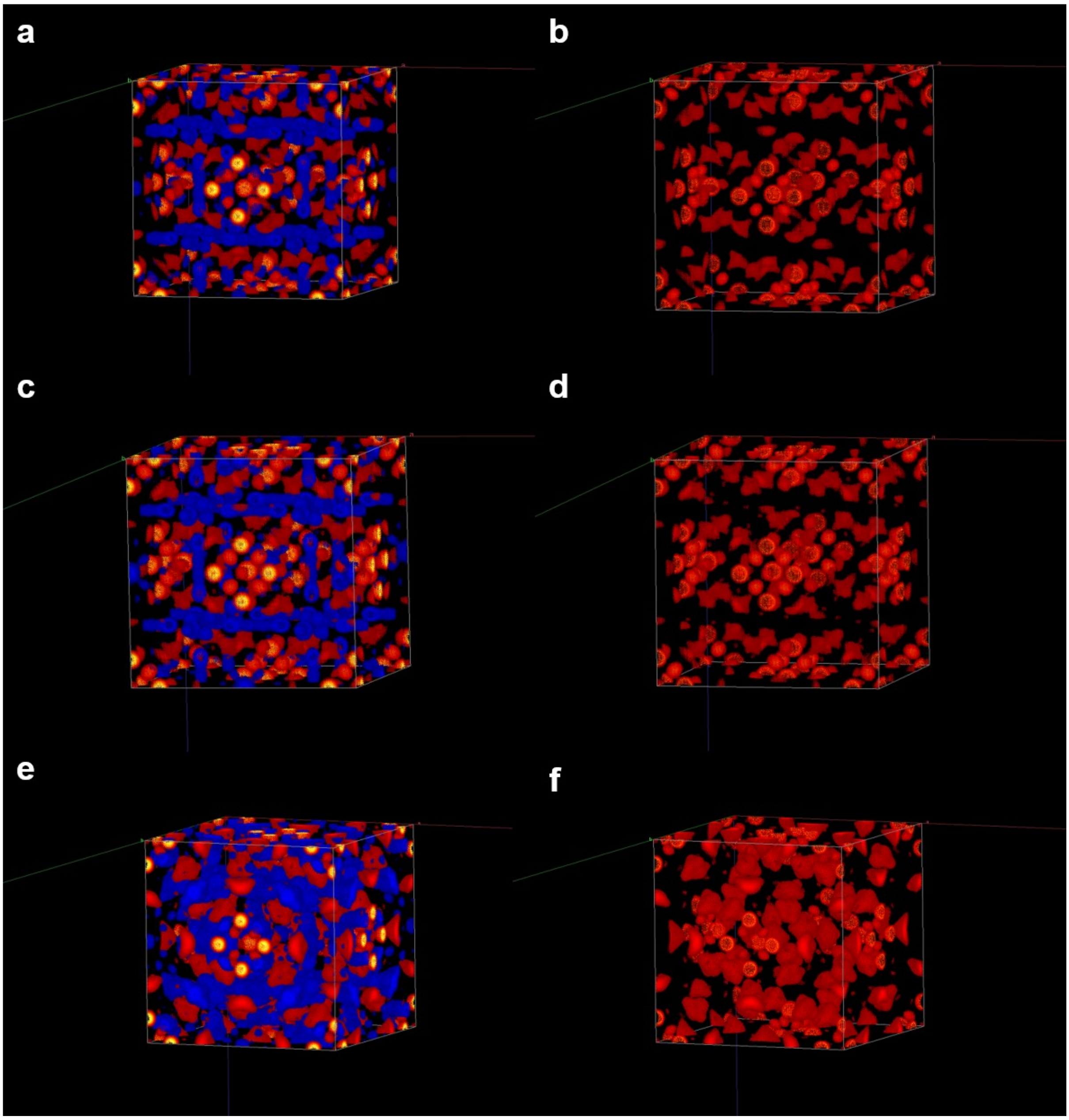

Figure S26. The Fourier electron difference maps of 1CuO-UiO-66-NH_2, 2CuO-UiO-66-NH_2, and 3CuO-UiO-66-NH_2.

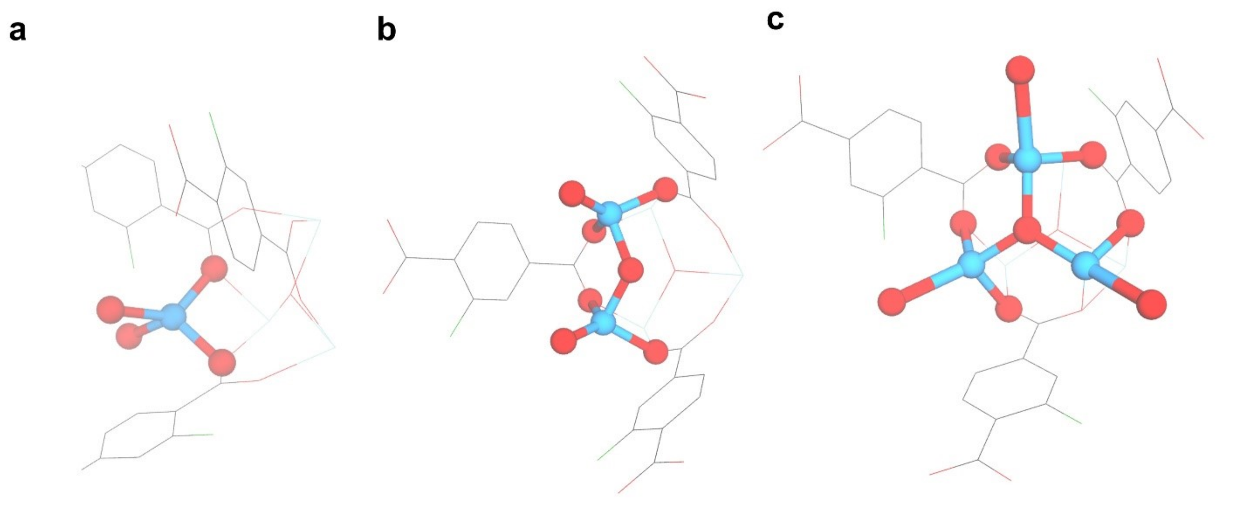

Figure S27. A clearer illustration of the Cu-oxo species/clusters in (a) **1CuO**, (b) **2CuO**, and (c) **3CuO**. Same colour scheme as applied.

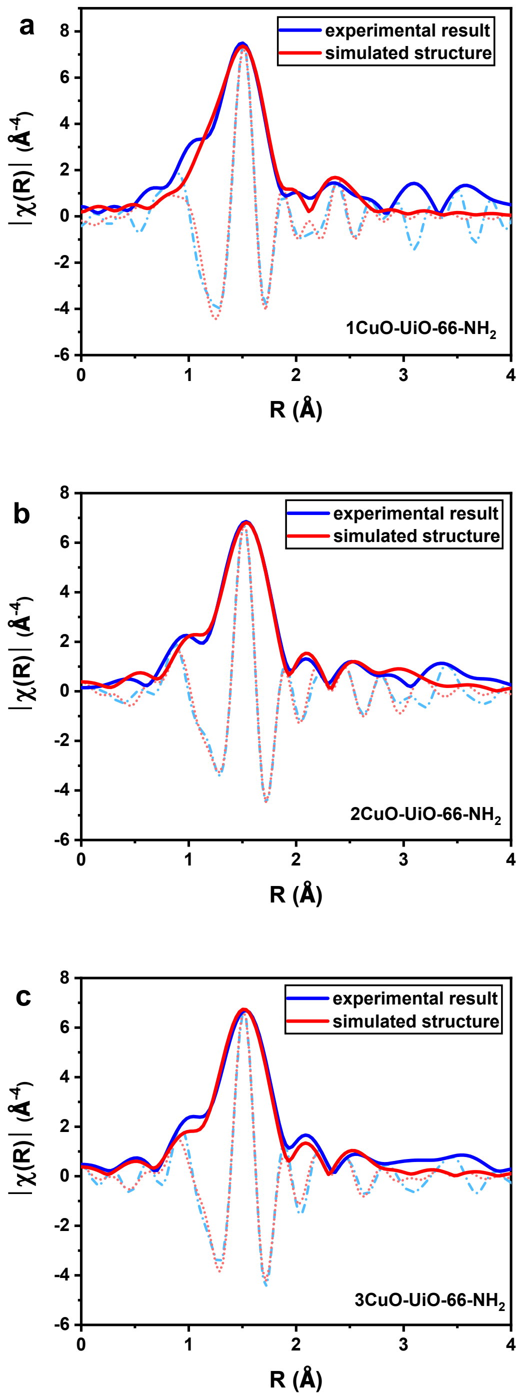

Figure S28. Fourier-transformed magnitude R-space of the experimental Cu K-edge k^3-weighted EXAFS. Experimental results (blue) and simulated data from Rietveld refined crystal structures (red) of (a) 1CuO-, (b) 2CuO-, and (c) 3CuO-UiO-66-NH_2.

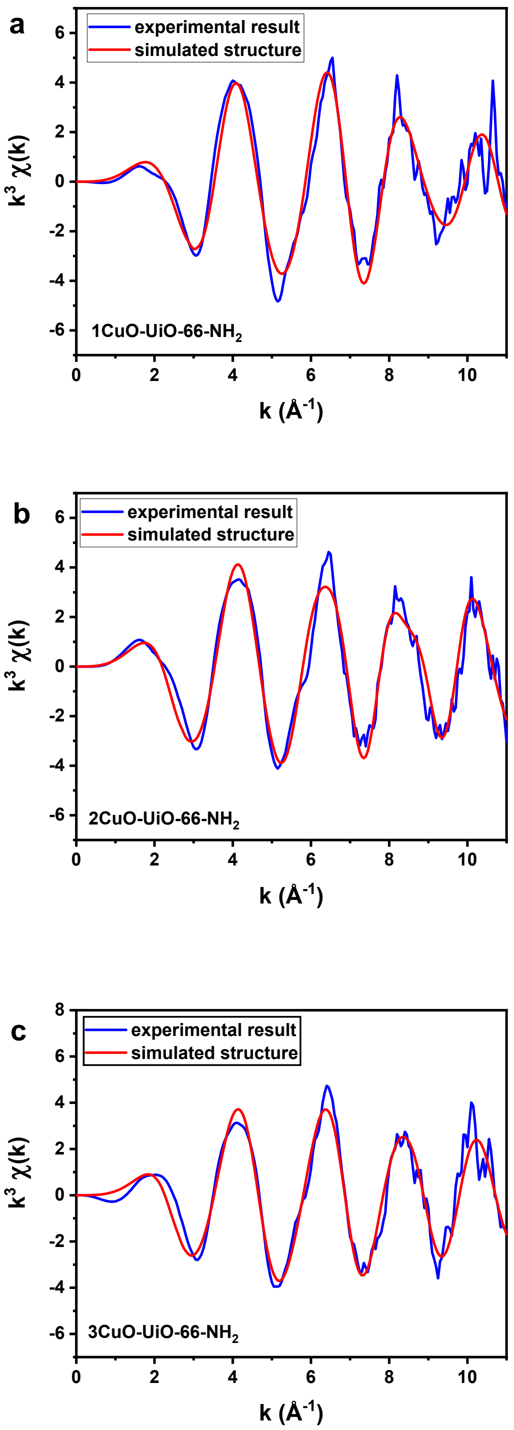

Figure S29. k-space of the experimental Cu K-edge k^3-weighted EXAFS: experimental results (blue) and simulated data from Rietveld refined crystal structures (red) of (a) 1CuO-, (b) 2CuO-, and (c) 3CuO-UiO-66-NH_2.

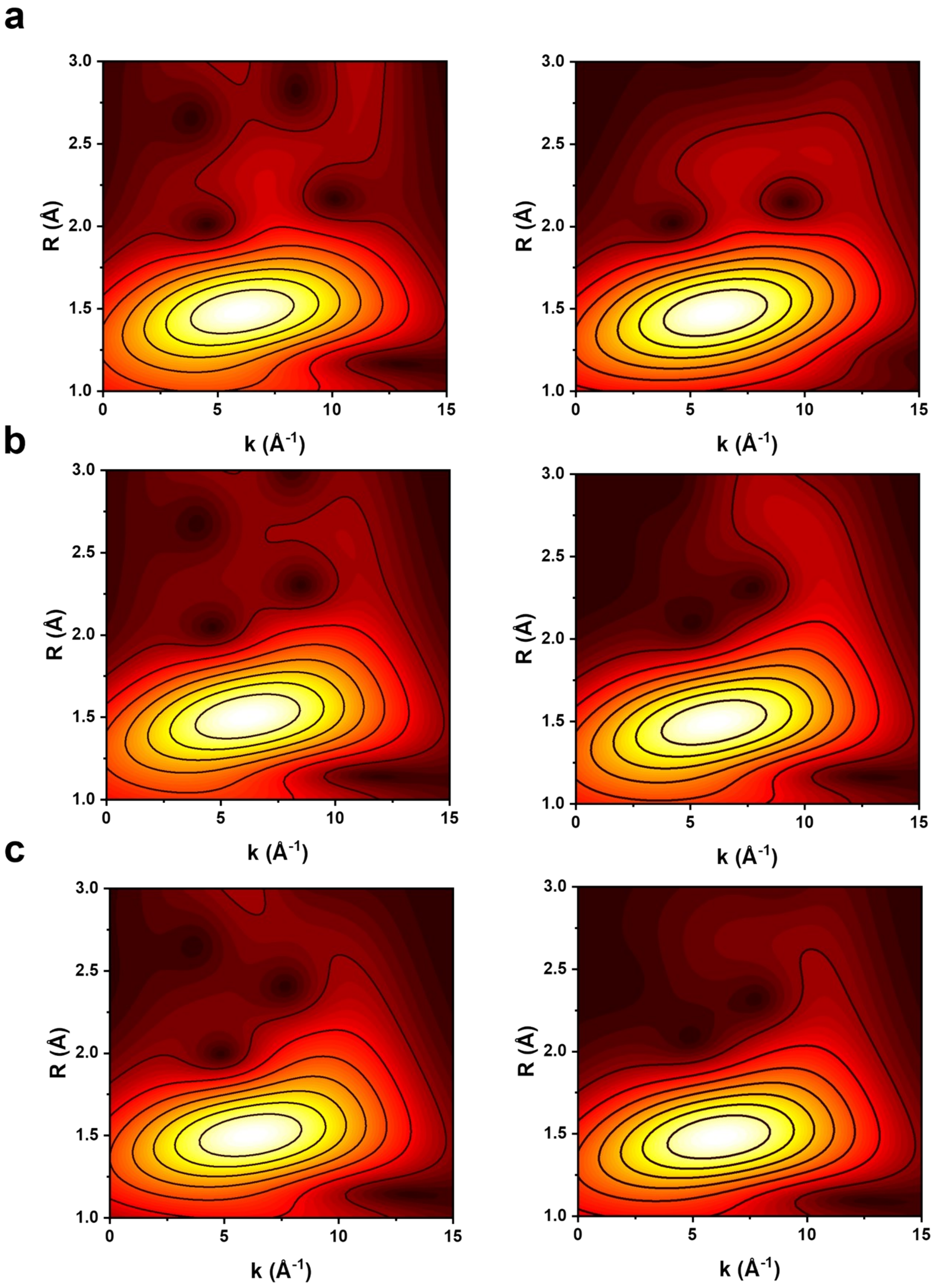

Figure S30. Comparison of WT-EXAFS. (left) Experimental data and (right) simulated profiles (a) 1CuO-, (b) 2CuO-, and (c) 3CuO-UiO-66-NH_2. WT-EXAFS profiles were generated using Morlet wavelet with $\kappa = 10$, $\sigma = 1$.

In the following, we compared the Rietveld refinement structures with the XAS experimental results and investigate the scattering contributions coming from different neighbouring atoms. The single scattering paths from the Cu absorber of all the samples include the Cu-O path in the first shell and the Cu-Zr path in the second shell. However, 2CuO-UiO-66-NH_2 and 3CuO-UiO-66-NH_2 contain Cu-Cu paths in the second shell. Because Cu has a much higher electron density than C, O, and N, Cu has a much greater influence on the EXAFS profiles.

Cu 1st and 2nd shell bonding information for the simulated EXAFS profiles (k-space (Figure S27), R-space (Figure S28) and WT (Figure S29)), using Morlet wavelet with κ = 10, σ = 1.

1CuO-UiO-66-NH_2: Cu-O_{fw} 1.939 Å (CN = 2), Cu-O_{ex} 1.980 Å (CN = 1), Cu…Zr 2.969 Å (CN = 1)

2CuO- and 3CuO-UiO-66-NH_2: The backscattering from Cu…Cu 3.351 Å (CN = 1) and Cu...Cu 3.370 Å (CN = 2) are also considered.

It can be seen that all simulated EXAFS profiles match well with the experimental data, which verifies the reliability of our Rietveld refinements.

In addition, as seen in the simulated WT-EXAFS profiles, the 1st shell coordination primarily contributes to the lobe at around R = 1.48 Å and k = 6.5 Å^{-1}. The stretched lobes as observed in the simulated WT-EXAFS profiles are originated from the 2nd shell Cu-Cu interactions.

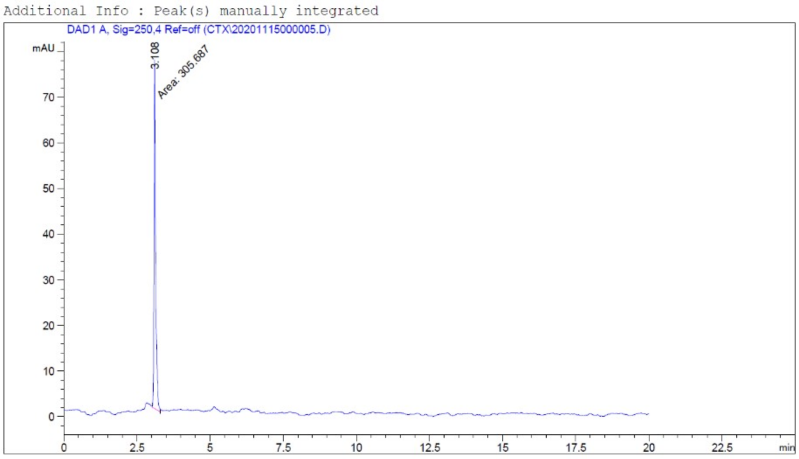

Figure S31. High-performance liquid chromatography of the liquid phase product after catalysis by 2CuO-UiO-66-NH_2. Only the peak of HCOOH was observed.

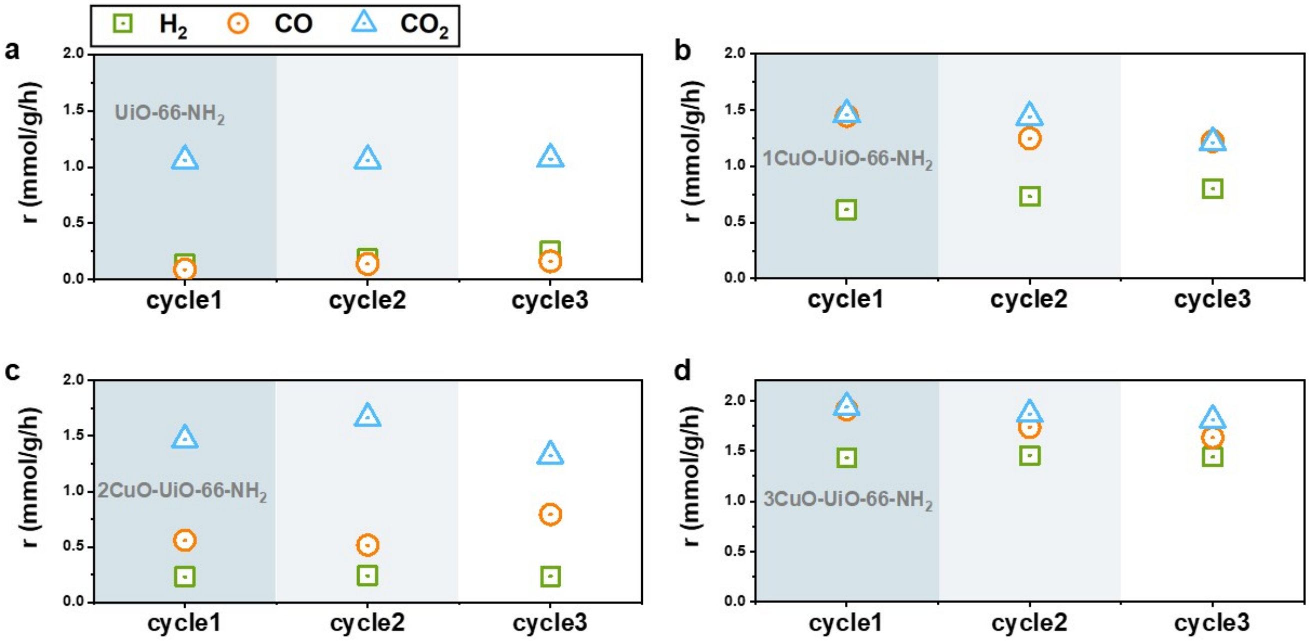

Figure S32. Stability study of (a) UiO-66-NH$_2$, (b) 1CuO-UiO-66-NH$_2$, (c) 2CuO-UiO-66-NH$_2$, and (d) 3CuO-UiO-66-NH$_2$.

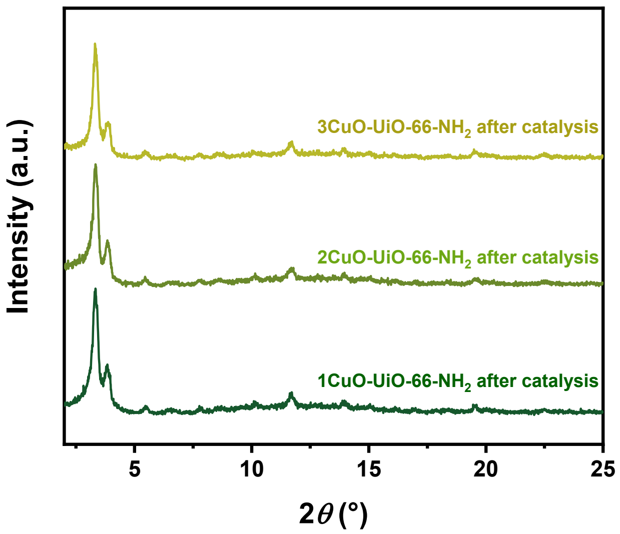

Figure S33. PXRD patterns of 1CuO-UiO-66-NH_2, 2CuO-UiO-66-NH_2, and 3CuO-UiO-66-NH_2 after catalysis for 3 cycles.

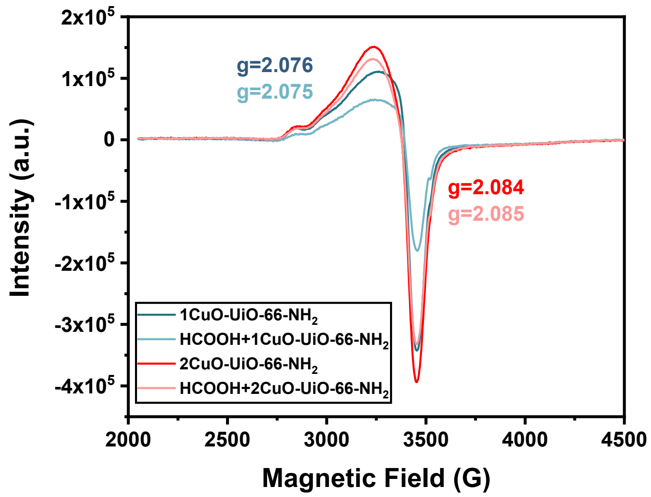

Figure S34. EPR spectra of 1CuO-UiO-66-NH_2 and 2CuO-UiO-66-NH_2 with and without pre-adsorbed FA.

Table S1. Elemental analysis: ICP-OES data of 1Cu^{2+}-UiO-66-NH_2, 2Cu^{2+}-UiO-66-NH_2, and 3Cu^{2+}-UiO-66-NH_2.

	Cu (ppm)	Zr (ppm)	Atomic ratio Cu(mmol):Zr(mmol)
1Cu^{2+}-UiO-66-NH_2	0.7478	11.7760	0.09:1
2Cu^{2+}-UiO-66-NH_2	1.0406	6.8777	0.21:1
3Cu^{2+}-UiO-66-NH_2	1.0529	5.0143	0.30:1

Table S2a. Fitting parameters for Cu K-edge EXAFS for 1Cu^{2+}-UiO-66-NH_2

Scattering Path	Coordination number	R/Å	σ^2
Cu-N	4.3(4)	1.98(1)	0.005(1)

S_0^2 was fixed at 0.867 and enot was returned to -4.86 ± 1.12 eV. Data range 3 ≤ k ≤ 10 Å^{-1}, 1.0 ≤ R ≤ 3.0 Å. 4 variable parameters used; 8.6875 independent data points; R factor = 0.80%.

Table S2b. Fitting parameters for Cu K-edge EXAFS for 2Cu^{2+}-UiO-66-NH_2

Scattering Path	Coordination number	R/Å	σ^2
Cu-N	4.8(9)	1.98(2)	0.006(3)

S_0^2 was fixed at 0.863 and enot was returned to -3.54 ± 2.17 eV. Data range 3 ≤ k ≤ 10 Å^{-1}, 1.0 ≤ R ≤ 3.0 Å. 4 variable parameters used; 8.6875 independent data points; R factor = 2.98%.

Table S2c. Fitting parameters for Cu K-edge EXAFS for 3Cu^{2+}-UiO-66-NH_2

Scattering Path	Coordination number	R/Å	σ^2
Cu-N	4.4(6)	2.00(1)	0.006(2)

S_0^2 was fixed at 0.863 and enot was fixed at -2.5 eV. Data range 3 ≤ k ≤ 10 Å^{-1}, 1.0 ≤ R ≤ 2.0 Å. 3 variable parameters used; 4.34375 independent data points; R factor = 0.57%

Table S3. Crystallographic parameters derived from the SXRD measurements of $1Cu^{2+}$-, $2Cu^{2+}$-, and $3Cu^{2+}$-UiO-66-NH_2.

	**UiO-66-NH_2**	**$1Cu^{2+}$-UiO-66-NH_2**	**$2Cu^{2+}$-UiO-66-NH_2**	**$3Cu^{2+}$-UiO-66-NH_2**
Diffractometer	MYTHEN	MYTHEN	MYTHEN	MYTHEN
X-ray source	SPring-8 BL02B2	SPring-8 BL02B2	SPring-8 BL02B2	SPring-8 BL02B2
Energy	18 keV	18 keV	18 keV	18keV
Wavelength (Å)	0.688213(2)	0.688213(2)	0.688213(2)	0.688213(2)
2θ - zero point (°)	-0.000106(2)	-0.000106(2)	-0.000106(2)	-0.000106(2)
Space group	*Fm-3m*	*Fm-3m*	*Fm-3m*	*Fm-3m*
Crystal system	Cubic	Cubic	Cubic	Cubic
a (Å)	20.78902(46)	20.80368(18)	20.76642(74)	20.8115(10)
V ($Å^3$)	8984.66(59)	9003.69(24)	8955.40(96)	9013.8(13)
2θ range for refinement (°)	2-30	2-30	2-30	2-30
Refinement methods	Rietveld	Rietveld	Rietveld	Rietveld
$R_{wp}/R_{exp}/R_p$ (%)	2.258/0.777/1.553	1.322/0.695/1.024	2.150/0.785/1.500	2.293/0.744/1.510
gof	2.905	1.903	2.741	3.079

R_{wp}: weighted profile; R_{exp}: expected; R_p: profile; *gof:* goodness-of-fit.

Table S4a. Fitting parameters for Cu K-edge EXAFS for 1CuO-UiO-66-NH_2.

Scattering Path	Coordination number	R/Å	σ^2
Cu-O	4.1(7)	1.94(1)	0.006(2)

Scale factor was fixed at 0.848 and enot = -2.54 ± 1.97eV. Data range 2 ≤ k ≤ 11 $Å^{-1}$, 1.0 ≤ R ≤ 2.1 Å. R factor = 1.28%. All Cu-O scattering paths were generated using monoclinic CuO (space group *C2/c*).

Table S4b. Fitting parameters for Cu K-edge EXAFS for 2CuO-UiO-66-NH_2.

Scattering Path	Coordination number	R/Å	σ^2
Cu-O	2.8(6)	1.96(1)	0.003(2)

Scale factor was fixed at 0.848 and enot = -0.80 ± 2.51 eV. Data range 2 ≤ k ≤ 11 $Å^{-1}$, 1.1 ≤ R ≤ 2.1 Å. R factor = 1.56%

Table S4c. Fitting parameters for Cu K-edge EXAFS for 3CuO-UiO-66-NH_2.

Scattering Path	Coordination number	R/Å	σ^2
Cu-O	2.9(6)	1.95(1)	0.003(2)

Scale factor was fixed at 0.848 and enot = -0.63 ± 2.20 eV. Data range 2 ≤ k ≤ 11 $Å^{-1}$, 1.1 ≤ R ≤ 2.1 Å. R factor = 1.22%.

Table S4d. Fitting parameters for Cu reference.

Scattering Path	Coordination number	R/Å	σ^2
Cu-O	12 (fixed)	2.53(1)	0.008(5)

Scale factor = 0.848 ± 0.049 and enot = 3.48 ± 0.61 eV. Data range 3 ≤ k ≤ 14 $Å^{-1}$, 1 ≤ R ≤ 3 Å. R factor = 0.55%. The calculated scale factor was used for the subsequent fitting analyses.

Table S5. The quantitative peak parameters of samples from SXRD.

	**UiO-66-NH_2**	**1CuO**	**2CuO**	**3CuO**
Peak asymmetry parameter	0.0001 (very symmetrical)	0.0001	0.0001	0.0001
LVol-Integral breadths	43.9(9)	44.1(3)	48.9(5)	45.8(4)
LVol-FWHM	61.3(10)	59.5(4)	68.4(5)	63.7(6)
Microstrain (e_0) *	0	0	0	0
Strain (Gaussian)	0	0.137(1)	0	0.166(2)
Strain (Lorentzian)	0.23(2)	0.15(1)	0.22(1)	0.24(1)

LVol - Volume weighted mean column heights
e_0 - dislocations, vacancies, and other defects, calculated from FWHMs.

Table S6. Crystallographic parameters from the SXRD measurements of UiO-66-NH_2, 1CuO-, 2CuO-, and 3CuO-UiO-66-NH_2.

	**UiO-66-NH_2**	**1CuO-UiO-66-NH_2**	**2CuO-UiO-66-NH_2**	**3CuO-UiO-66-NH_2**
Diffractometer	MYTHEN	MYTHEN	MYTHEN	MYTHEN
X-ray source	SPring-8 BL02B2	SPring-8 BL02B2	SPring-8 BL02B2	SPring-8 BL02B2
Energy	18 keV	18 keV	18 keV	18keV
Wavelength (Å)	0.688213(2)	0.688213(2)	0.688213(2)	0.688213(2)
2θ - zero point (°)	-0.000106(2)	-0.000106(2)	-0.000106(2)	-0.000106(2)
Space group	*Fm-3m*	*Fm-3m*	*Fm-3m*	*Fm-3m*
Crystal system	Cubic	Cubic	Cubic	Cubic
a (Å)	20.78902(46)	20.80368(18)	20.80197(79)	20.79361(37)
V (Å^3)	8984.66(59)	9003.69(24)	9001.48(27)	8990.62(48)
2θ range for refinement (°)	2-30	2-30	2-30	2-30
Refinement methods	Rietveld	Rietveld	Rietveld	Rietveld
$R_{wp}/R_{exp}/R_p$ (%)	2.258/0.777/1.553	1.322/0.695/1.024	1.407/0.735/1.108	2.293/0.744/1.510
gof	2.905	1.903	1.914	3.079

R_{wp}: weighted profile; R_{exp}: expected; R_p: profile; *gof:* goodness-of-fit.

Table S7. Comparison of the peak intensities of **1CuO**, **2CuO**, and **3CuO** (normalised using (200)).

h	*k*	*l*	1CuO	2CuO	3CuO
1	1	1	2.72854	2.69248	2.71549
2	0	0	**1**	**1**	**1**
2	2	0	0.29677	0.30904	0.30948
3	1	1	0.17851	0.19325	0.19027
2	2	2	0.25366	0.25330	0.24354
4	0	0	0.43363	0.40181	0.39735
3	3	1	0.30680	0.32718	0.31791
4	2	0	0.39161	0.36579	0.35217
4	2	2	0.24963	0.26901	0.25025
3	3	3	0.39762	0.41282	0.3939
5	1	1	0.39762	0.41282	0.3939
4	4	0	0.22488	0.24792	0.23321
5	3	1	0.72774	0.73362	0.74817
4	4	2	1.60786	1.60868	1.57285
6	0	0	1.60786	1.60868	1.57285
6	2	0	0.03611	0.04327	0.03388
5	3	3	0.36014	0.34394	0.3474
6	2	2	0.12500	0.14752	0.13462
4	4	4	0.67093	0.70631	0.71037

Table S8. Comparison of the R-factors of the fittings with and without the inclusion of the Cu-oxo species.

	**Without Cu-oxo species**			**With Cu-oxo species**		
	R_{wp}	R_{exp}	gof	R_{wp}	R_{exp}	gof
**1CuO**	4.00	0.69	5.74	1.32	0.69	1.90
**2CuO**	4.71	0.73	6.41	1.41	0.74	1.91
**3CuO**	3.61	0.68	5.33	2.29	0.74	3.08

Table S9a. Atomic parameters derived from the Rietveld refinement of the SXRD measurement of 1CuO-UiO-66-NH_2, measured at 25 °C.

Species	Atom	*x*	*y*	*z*	SOF	B_{eq} (Å^2)	Wyckoff letter
MOF-host	Zr1	-0.1269	0	0	1	0.6	*d*
	O2	0.0576	0.0576	0.0576	1	2	*f*
	C1	0.15723	0	0.15723	1	4	*h*
	C2	0.208554	0	0.208554	1	4	*h*
	C3	0.272165	0	0.19151	1	4	*j*
	O1	0.173204	0	0.097613	1	4	*j*
	N1	0.28896	0	0.128829	0.25	4	*j*
Extra-framework site	Cu1	0.0726	0.2259	0.0726	$0.10^{\#}$	5	*l*
	OCu1	0.129	0.129	0.278	$0.10^{\#}$	5	*l*
	OCu2	0.05	0.05	0.315	$0.10^{\#}$	5	*l*

SOF: site occupancy factor; B_{eq}: isotropic displacement factor. # The site occupancy factors of the Cu site(s) are fixed based on elemental analysis.

Table S9b. Atomic parameters derived from the Rietveld refinement of the SXRD measurement of 2CuO-UiO-66-NH_2, measured at 25 °C.

Species	Atom	*x*	*y*	*z*	SOF	B_{eq} (Å^2)	Wyckoff letter
MOF-host	Zr1	0.1266	0	0	1	0.6	*d*
	O2	0.0632	0.0632	0.0632	1	2	*f*
	C1	0.15679	A	0.15679	1	4	*h*
	C2	0.208138	0	0.208138	1	4	*h*
	C3	0.271778	0	0.191086	1	4	*j*
	O1	0.172772	0	0.097145	1	4	*j*
	N1	0.288582	0	0.128375	0.25	4	*j*
Extra-framework site	Cu1	0.0910	0.2048	0.0910	$0.10^{\#}$	5	*l*
	Cu2	0.2048	0.0910	0.7950	$0.10^{\#}$	5	*l*
	OCu1	0.130	0.130	0.120	$0.10^{\#}$	5	*l*
	OCu2	0.129	0.129	0.278	$0.10^{\#}$	5	*l*
	OCu3	0.278	0.129	0.871	$0.10^{\#}$	5	*l*

SOF: site occupancy factor; B_{eq}: isotropic displacement factor. # The site occupancy factors of the Cu site(s) are fixed based on elemental analysis.

Table S9c. Atomic parameters derived from the Rietveld refinement of the SXRD measurement of 3CuO-UiO-66-NH_2, measured at 25 °C.

Species	Atom	*x*	*y*	*z*	SOF	B_{eq} (Å^2)	Wyckoff letter
MOF-host	Zr1	0.1285	0	0	1	0.6	*d*
	O2	0.0597	0.0597	0.0597	1	2	*f*
	C1	0.15679	0	0.15679	1	4	*h*
	C2	0.208139	0	0.208139	1	4	*h*
	C3	0.271779	0	0.191086	1	4	*j*
	O1	0.172772	0	0.097145	1	4	*j*
	N1	0.288582	0	0.128375	0.25	4	*j*
Extra-framework site	Cu1	0.0910	0.2056	0.9090	$0.10^{\#}$	5	*l*
	Cu2	0.2056	0.0910	0.9090	$0.10^{\#}$	5	*l*
	Cu3	0.0910	0.0910	0.7944	$0.10^{\#}$	5	*l*
	OCu1	0.130	0.130	0.130	$0.10^{\#}$	5	*l*
	OCu2	0.0440	0.0440	0.3120	$0.10^{\#}$	5	*l*
	OCu3	0.0440	0.3120	0.9560	$0.10^{\#}$	5	*l*
	OCu4	0.3120	0.0440	0.9560	$0.10^{\#}$	5	*l*

SOF: site occupancy factor; B_{eq}: isotropic displacement factor. # The site occupancy factors of the Cu site(s) are fixed based on elemental analysis.

References

[1] Q. Xue, Y. Xie, S. Wu, T. S. Wu, Y. L. Soo, S. Day, C. C. Tang, H. W. Man, S. T. Yuen, K. Y. Wong, Y. Wang, B. T. W. Lo, S. C. E. Tsang, *Nanoscale* **2020**, *12*, 23206.
[2] P. Thompson, D. E. Cox, J. B. Hastings, *J. Appl. Crystallogr.* **1987**, *20*, 79.
[3] D. Watkin, *Acta Crystallogr. Sect. B Struct. Sci.* **2000**, *56*, 747.
[4] R. I. Cooper, A. L. Thompson, D. J. Watkin, *J. Appl. Crystallogr.* **2010**, *43*, 1100.
[5] H. Van Koningsveld, *Acta Crystallogr. Sect. B Struct. Sci.* **1990**, *46*, 731.
[6] B. F. Mentzen, *Mater. Res. Bull.* **1987**, *22*, 489.
[7] R. Goyal, A. N. Fitch, H. Jobic, *J. Phys. Chem. B* **2000**, *104*, 2878.
[8] B. Ravel, M. Newville, in *J. Synchrotron Radiat.*, International Union Of Crystallography, **2005**, pp. 537–541.
[9] H. Funke, A. C. Scheinost, M. Chukalina, *Phys. Rev. B - Condens. Matter Mater. Phys.* **2005**, *71*, 094110.
[10] S. J. Clark, M. D. Segall, C. J. Pickard, P. J. Hasnip, M. I. J. Probert, K. Refson, M. C. Payne, *Zeitschrift fur Krist.* **2005**, *220*, 567.
[11] J. P. Perdew, K. Burke, M. Ernzerhof, *Phys. Rev. Lett.* **1996**, *77*, 3865.
[12] J. P. Perdew, J. A. Chevary, S. H. Vosko, K. A. Jackson, M. R. Pederson, D. J. Singh, C. Fiolhais, *Phys. Rev. B* **1992**, *46*, 6671.
[13] J. D. Head, M. C. Zerner, *Chem. Phys. Lett.* **1985**, *122*, 264.
[14] M. I. J. Probert, M. C. Payne, *Phys. Rev. B - Condens. Matter Mater. Phys.* **2003**, *67*, 075204.
[15] P. J. Hasnip, C. J. Pickard, *Comput. Phys. Commun.* **2006**, *174*, 24.
[16] S. Kawaguchi, K. Sugimoto, *Hoshako* **2018**, *31*, 333.
[17] B. T. W. Lo, L. Ye, J. Qu, J. Sun, J. Zheng, D. Kong, C. A. Murray, C. C. Tang, S. C. E. Tsang, *Angew. Chem. Int. Ed.* **2016**, *55*, 5981.
